# Quality and composition of Albendazole, Mebendazole and Praziquantel available in Burkina Faso, Côte d’Ivoire, Ghana and Tanzania

**DOI:** 10.1371/journal.pntd.0009038

**Published:** 2021-01-25

**Authors:** Moritz Seitzer, Sylvia Klapper, Humphrey D. Mazigo, Ulrike Holzgrabe, Andreas Mueller

**Affiliations:** 1 Medical Mission Institute, Würzburg, Bavaria, Germany; 2 Institute of Pharmacy and Food Chemistry, Julius-Maximilians-University of Würzburg, Würzburg, Germany; 3 Department of Medical Parasitology and Entomology, Catholic University of Health and Allied Sciences, Mwanza, Lake Zone, United Republic of Tanzania; 4 Department of Tropical Medicine, Klinikum Würzburg Mitte gGmbH (Medical Mission Hospital), Würzburg, Germany; Ministère de la Santé Publique et de la Lutte contre les Endémies, NIGER

## Abstract

**Background:**

Even though the international combat against Neglected Tropical Diseases such as schistosomiasis or soil-transmitted helminthiases depends on reliable therapeutics, anthelminthic pharmacovigilance has been neglected on many national African drug markets. Therefore, quality and composition of Albendazole, Mebendazole and Praziquantel locally collected in Burkina Faso, Côte d’Ivoire, Ghana and Tanzania were analysed.

**Methods:**

Samples of 88 different batches were obtained from randomly selected facilities. Sampling took place in Northwest Tanzania, Western Burkina Faso, Southeast Côte d’Ivoire and Southwest Ghana. Visual examination of both packaging and samples was performed according to the WHO ‘Be Aware’ tool. Products were then screened with the GPHF Minilab, consisting of tests of mass uniformity, disintegration times and thin-layer chromatography (TLC). Confirmatory tests were performed according to international pharmacopoeiae, applying assays for dissolution profiles and high-performance liquid chromatography (HPLC).

**Findings:**

Despite minor irregularities, appearance of the products did not hint at falsified medicines. However, 19.6% of the brands collected in Ghana and Tanzania were not officially licensed for sale. Mass uniformity was confirmed in 53 out of 58 brands of tablets. 41 out of 56 products passed disintegration times; 10 out of the 15 failing products did not disintegrate at all. Evaluating TLC results, only 4 out of 83 batches narrowly missed specification limits, 18 batches slightly exceeded them. Not more than 46.3% (31 / 67) of the tablets assayed passed the respective pharmaceutical criteria for dissolution. HPLC findings confirmed TLC results despite shifted specification limits: 10 out of 83 tested batches contained less than 90%, none exceeded 110%.

**Conclusion:**

In the four study countries, no falsified anthelminthic medicine was encountered. The active pharmaceutical ingredient was not found to either exceed or fall below specification limits. Galenic characteristics however, especially dissolution profiles, revealed great deficits.

## Author summary

Among Neglected Tropical Diseases, schistosomiasis and soil-transmitted helminthiases are still highly prevalent and affect more than 1.5 billion people on our planet. Key players in the combat against these entities are the anthelminthic medicines Albendazole, Mebendazole and Praziquantel, which are applied in therapy and preventive chemotherapy likewise. Despite widely available in tropical regions and, particularly for both benzimidazoles, relatively cheap, their quality has been poorly monitored. By this study, we illuminated products from East (Tanzania) and West Africa (Burkina Faso, Côte d’Ivoire and Ghana) and thus extended the African map of reproducibly assessed deworming drugs. Although containing sufficient active pharmaceutical ingredient, a failure of more than 50% in galenic features significantly mars a thorough elimination of parasites from patients. Our findings emphasize the need for quality-assured products. Regular local screening, efficient and expressive confirmation of irregularities and a swift appropriate reaction by governmental authorities contribute to a successful fight against these infectious diseases. Otherwise, their sequelae pose a tremendous burden to the development and prosperity of afflicted regions–not only in heavily affected Africa but around the globe.

## Introduction

Succeeding the 8 Millennium Development Goals (MDGs) that had been resolved back in September 2000, 17 Sustainable Development Goals (SDGs) were framed in 2015 to terminate global inequality and poverty [1]. Herein, United Nation member states committed themselves to, inter alia, intensify the fight against climate change, pollution, injustice or gender / social / geographic inequality. Peace, well-being and equal rights were declared destinations up to 2030 that should ‘leave no one behind’. En route to improved health conditions, MDG 6 and SDG 3.3 lay special focus upon the combat against communicable diseases, which are found to a larger extent within the tropical regions than beyond them [2]. While infectious diseases like malaria, tuberculosis or the acquired immune deficiency syndrome are well known and targeted all over the world, 20 entities that are - unfortunately still applicably - summarized as Neglected Tropical Diseases (NTDs) seem to be confined to low- and middle-income countries not only geographically but also in the wide opinion of people not affected by them. However, approximately one and a half billion people in 149 countries - about one out of five global citizens - are potentially threatened by at least one of these NTDs [3,4]. Among them, helminthiases extensively contribute to both morbidity and mortality, which are caused by NTDs in affected regions. Soil-transmitted helminthiases (STH) and schistosomiasis, key players among NTDs, are responsible for a combined impact of almost eight million disability-adjusted life years (data from 2015) [5]. Considering the fact that both diseases are found in tropical zones around the world and affect people of all ages, the World Health Organization (WHO) alongside numerous governmental and non-governmental organisations have been increasing their efforts in combatting these diseases [6–8]. Besides public health information and education, the most promising approaches to targeting these infections are extensive mass drug administration (MDA) of preventive chemotherapy to the most susceptible social strata [8–10] and the international campaign of water, sanitation and hygiene (WASH) to reduce breeding grounds, transmission and sequelae [11,12]. The drugs of choice in treatment of STH are Albendazole (ABZ) and Mebendazole (MBZ), while Praziquantel (PZQ) is routinely administered against schistosomiasis [7,8,13]. According to national and WHO action plans, these medicines are distributed (bi-)annually to (pre-)school children and people at risk as single dose of 400 mg of ABZ, 500 mg of MBZ and 40 mg per kg of PZQ [7] - the anthelminthics for preventive chemotherapy are donated under the London Declaration of 2012 by international pharmaceutical companies [9]. However, preventive chemotherapy does not regularly reach all risk groups outside school. Since public knowledge of typical and striking symptoms of schistosomiasis and STH is not ubiquitous, many patients are prone to not being treated appropriately [14]. Should they finally be advised to try anthelminthic treatment after a prolonged course of disease, they have to rely on products locally available. Unlike in countries not experiencing these NTDs on a regular base, many disparate brands of especially ABZ and MBZ can be found on local markets, which are manufactured in different parts of the world. In order to successfully fight such NTDs, periodic quality assessment (QA) of the medicines is necessary. SDG 3.8 strengthens an universal access to quality-assured medical and pharmaceutical products [2]. Basic governmental structures, weak law enforcement and confusing drug supply chains however regularly limit effective pharmacovigilance programs [15].

In Africa, data on the quality of anthelminthic drugs is sparse. Drug quality in Ethiopia was repeatedly determined to be of predominantly decent content but variable composition [16–19]. From other countries throughout the continent, only individual batches were analysed to point out on mostly acceptable concentrations of active pharmaceutical ingredient (API) [20–22]. As schistosomiasis and STH are endemic to countries within the drainage basin of large water bodies like Lake Victoria and Lake Volta, a reliable overview on the local variety of therapeutics is paramount. Herein, Burkina Faso (BF), Côte d’Ivoire (CI), Ghana (GH) and Tanzania (TZ) are still facing a large public burden of both NTDs. By applying the strategies mentioned above, large quantities of anthelminthic medicines are used to combat NTDs–and thus potentially attract questionable drug manufacturers implementing low-quality medicines. Tackling this threat, we aimed at assessing the quality and the composition of ABZ, MBZ and PZQ available in these four countries to determine the risk of coming across substandard and / or falsified (SF) anthelminthic medicines.

## Materials and methods

### Visual examination

Physical characteristics of the products and their packages were initially evaluated, applying the WHO ‘Be Aware’ tool [23]. Checks of the packages were systematically performed for suitability of the container, an enclosed leaflet and adequate labelling indicating trade and / or brand name, name of the API, the manufacturer’s name, logo and address, dosage form and statement, medicine strength, number of units per container, batch number, manufacturing and expiring dates as well as storage information. Tablets were screened for uniformity of colour, shape, size and texture, markings, breaks / cracks / splits, embedded surface spots or contamination and smell (not appropriate for suspensions).

### Quality assessment applying the GPHF Minilab

#### Chemicals and reference substances

Reference standards for TLC containing the respective APIs were purchased from GPHF. For testing in Tanzania, the following chemicals were purchased: acetic acid 100% (Val de Reuil, France), acetone and ammonia 25% solution from Labtech Chemicals (Tanzania), ethyl acetate and toluene from Loba Chemie (Mumbai, India) and methanol from Trust Chemical Laboratories (Tanzania). In Germany, acetic acid 100%, acetone, ammonia 25% solution, sulfuric acid 96% and toluene were purchased from Merck (Darmstadt, Germany). Acetic acid 96%, ethyl acetate and methanol were obtained from Carl Roth (Karlsruhe, Germany).

#### Mass uniformity

20 randomly selected tablets per product (or batch, when collected enough) were weighed on a calibrated analytical balance (Kern CM Version 1.8 in Tanzania and Kern EMB 600–2 in Germany, both by Kern & Sohn GmbH, Germany). Whenever less tablets had been obtained, the respective proportions of 90% (instead of 18 tablets) and 10% (instead of two tablets) were calculated.

#### Disintegration times

Six tablets per batch (or product, when necessary), chosen at random, were tested according to the basic Minilab kit consisting of jars, a thermometer and a portable heating plate. Criteria of this method were marked as passed when samples placed into a vessel containing 100 mL of warm tap water at approximately 37°C fully disintegrated within 30 minutes. Whenever a firm core was still discernible after that time, measuring was continued. Tests were terminated after 45 minutes even if the size of the tablets had not significantly decreased by then. Batches varying distinctively from each other were listed separately.

#### Thin-layer chromatography

TLCs were performed according to the Minilab manual [24], using aluminium chromatoplates pre-coated with silica gel 60 F_254_ (size of 5 x 10 cm; by Merck, Darmstadt, Germany). API content was determined semi-quantitatively against a standard solution concentrated to a 80% lower specification limit (LSL) and 100% upper specification limit (USL) of the declared amount, respectively.

ABZ were diluted with acetic acid 96% to 1.25 mg/mL without pre-treatment. MBZ were converted into suspension by adding 1 mL (for a 100 mg give) or 2 mL (for a 500 mg give) of purified water and then diluted with acetic acid 96% to a concentration of 1.25 mg/mL. The mobile phase was composed of 14 mL of toluene, 4 mL of ethyl acetate and 4 mL of either acetic acid 100% (for MBZ) or acetic acid 96% (for ABZ). To increase the resolution between ABZ and MBZ, a second mobile phase consisting of 27 mL ethyl acetate, 3 mL methanol and 5 mL ammonia solution 25% was applied.

PZQ tablets were dissolved and diluted with methanol to a concentration of 15 mg/mL. The respective mobile phase consisted of 14 mL of acetone and 7 mL of toluene.

All APIs were examined under 254 nm UV light. Afterwards, ABZ and PZQ were stained by iodine vapour. MBZ were additionally treated with methanolic sulfuric acid and detected under 366 nm UV light.

### High-performance liquid chromatography

#### Chemicals and reference substances

All reagents were of analytical grade. Ammonium acetate, Mebendazole and Oxibendazole were purchased from Sigma-Aldrich Chemie GmbH (Steinheim, Germany). Sulphuric acid 95–97% was obtained from Fisher Scientific (Loughborough, United Kingdom), hydrochloric acid 37% from Bernd Kraft GmbH (Duisburg, Germany), formic acid 99% from Grüssing (Filsum, Germany) and both HPLC grade acetonitrile and HPLC grade methanol from VWR International GmbH (Darmstadt, Germany). Albendazole was bought from Fagron GmbH (Barsbüttel, Germany), Praziquantel CRS and Praziquantel for system suitability CRS from EDQM (Strasbourg, France). Water for HPLC was purified using a Milli-Q purification system by Merck Millipore (Schwalbach, Germany).

#### Apparatus

HPLC experiments were performed on an Agilent 1100 modular chromatographic system (Agilent technologies, Waldbronn, Germany) consisting of a vacuum degasser (G1379A), a binary pump (G1312A), an autosampler (G1313A), a thermostatted column compartment (G1316A) and a diode array detector (G1314A). Agilent ChemStation Rev B.03.02 software was used for data processing.

#### Methods

The HPLC-UV methods applied in the determination of the content of ABZ, MBZ and PZQ were related to the assays described in The International Pharmacopoeia 7 (Ph. Int. 7) [25] and the tests for related substances of the European Pharmacopoeia 9 (Ph. Eur. 9) [26], respectively. A five-point calibration was used for calculation.

#### Albendazole

For stock solution, 25.0 mg of ABZ reference substance were dissolved in 5 ml of a mixture of sulphuric acid and methanol (1:99, v/v) and 15 mL of methanol, and subsequently diluted with methanol to 25.0 mL. A volume of 1.0 mL, 1.5 mL, 2.0 mL, 2.5 mL and 3.0 mL of stock solution was diluted with methanol to 10.0 mL.

For sample solution, one randomly selected tablet - alternatively 5.0 mL of suspension - were dissolved in 5 mL of acidified methanol (sulphuric acid/methanol; 1:99, v/v) and 15 mL of methanol under sonication and subsequently diluted with methanol to 50.0 mL. According to the declared content, 1.25 mL, 2.0 mL and 5.0 mL of the solution were diluted to 50.0 mL with methanol obtaining a concentration of 0.2 mg/mL. The sample solution was filtered through a PTFE membrane (diameter 13 mm, pore size 0.2 μm) (VWR International GmbH, Darmstadt, Germany).

The content of ABZ was determined as described in Ph. Int. 7 [25]. An Hypersil ODS (C18) column (250 mm x 4.6 mm; particle size 5 μm; pore size 120 Å) (Thermo Fisher Scientific, Dreieich, Germany) was used as stationary phase. The mobile phase was composed of a solution of monobasic ammonium phosphate (1.67 g/L) and methanol (30:70, v/v). An injection volume of 20 μL, a flow rate of 0.7 mL/min and a detection wavelength of 254 nm were adjusted. Analyses were performed at room temperature.

#### Mebendazole

For stock solution, 100.0 mg of MBZ reference substance were dissolved in 30.0 mL of anhydrous formic acid, sonicated for 20 minutes and subsequently diluted with ultrapure water and methanol (40:60, v/v) to 100.0 mL. A volume of 2.0 mL, 3.0 mL, 5.0 mL, 7.0 mL and 8.0 mL of stock solution was diluted to 100.0 mL with ultrapure water and methanol (40:60, v/v).

For sample solution, one tablet - or 5.0 mL of suspension - was dissolved in 30.0 mL of anhydrous formic acid under 20 minutes of sonication and then diluted with ultrapure water and methanol (40:60, v/v) to 100.0 mL. Depending on the declared content of MBZ, 1.0 mL and 5.0 mL of the solution were diluted to 100.0 mL with ultrapure water and methanol (40:60, v/v) reaching a concentration of 0.05 mg/mL. The sample solution was filtered through a PTFE membrane.

The method used was in accordance with the assay described in Ph. Int. 7 [25] at room temperature. The stationary phase was a C18 YMC Pack Pro column (150 mm x 4.0 mm; particle size 3 μm; pore size 120 Å) (YMC, Dinslaken, Germany). A mixture of a solution of ammonium acetate (7.5 g/L) and acetonitrile (75:25, v/v) was used as mobile phase. An injection volume of 10 μL, a flow rate of 0.9 mL/min and a detection wavelength of 250 nm were set.

#### Praziquantel

For stock solution, 40.0 mg of PZQ reference substance were dissolved in 5.0 mL of a mixture of ultrapure water and acetonitrile (55:45, v/v). It was sonicated for 5 minutes and diluted with ultrapure water and acetonitrile (55:45, v/v) to 10.0 mL. 0.125 mL, 0.250 mL, 0.375 mL, 0.500 mL and 0.625 mL of stock solution were diluted with ultrapure water and acetonitrile (55:45, v/v) to 10.0 mL.

For sample solution, one tablet was mixed with 70.0 mL of a mixture of ultrapure water and acetonitrile (55:45, v/v), sonicated for 20 minutes to dissolve and then diluted with ultrapure water and acetonitrile (55:45, v/v) to 100.0 mL. 3.0 mL of the solution were diluted to 100.0 mL with ultrapure water and acetonitrile (55:45, v/v) and filtered through a PTFE membrane.

The chromatographic conditions were based on the HPLC method for the test for related substances, described in the monography of Praziquantel in Ph. Eur. 9 [26] with a detection wavelength of 210 nm. A Hypersil ODS (C18) column (250 mm x 4.6 mm; particle size 5 μm; pore size 120 Å) was used as stationary phase at room temperature. The eluent consisted of ultrapure water and acetonitrile (55:45, v/v). The injection volume was set at 20 μL, the flow rate at 1.15 mL/min.

### Dissolution

#### Chemicals

All reagents were of analytical grade. Sodium dodecyl sulphate (SDS) was purchased from Sigma-Aldrich Chemie GmbH (Steinheim, Germany), hydrochloric acid 37% from Bernd Kraft GmbH (Duisburg, Germany). Water for HPLC was purified using Milli-Q purification system by Merck Millipore (Schwalbach, Germany).

#### Apparatus

Dissolution analyses were performed on an Erweka Dissolutiontester DT 6 R (Erweka GmbH, Langen, Germany) with 6 dissolution vessels and paddles, corresponding to test apparatus 2 as described by the United States Pharmacopoeia and National Formulary 41 (USP-NF 41) [27].

#### Methods

Evaluating dissolution profiles, methods described in both Ph. Int. 7 [25] and USP-NF 41 [27] were adopted (Table 1). The amount of released API was determined by HPLC-UV (cf. chapter 2.3). 900 mL of dissolution medium were tempered at 37 ± 0.5°C. Rotation speed was set at 50 and 75 revolutions per minutes (rpm), respectively. At sampling time, a volume of 10.0 mL was withdrawn from the vessel and replaced by dissolution medium. When necessary, the sample was diluted with dissolution medium and subsequently passed through a cellulose acetate membrane (diameter 25 mm, pore size 0.45 μm) (VWR International GmbH, Darmstadt, Germany).

**Table 1:** Dissolution test conditions.

API	ABZ		MBZ			PZQ
Method	Ph. Int.		Ph. Int.	Ph. Int.	USP	USP
**API content [mg]**	200	400	100	500	100	600
**Dissolution medium**	0.1 M HCl		0.1 M HCl	0.01 M HCl + 1% SDS	0.1 M HCl + 1% SDS	0.1 M HCl + 2 mg/mL SDS
**Rotation speed [rpm]**	75		75	75	75	50
**Sampling time [min]**	10, 20, 30		30, 60, 90, 120	15, 30, 45, 60	30, 60, 90, 120	15, 30, 45, 60
**Dilution**	Undiluted	1:1	1:1	1:10	1:1	3:10
**Q [%]** *	80		60	70	75	75

* Q is the specified amount of dissolved API expressed as a percentage of the labelled content [25,27]

## Results

### Sample collection

Even though both schistosomiasis and STH are endemic to all four countries, differences were seen in the availability of ABZ, MBZ and PZQ. The two benzimidazoles, owing to low prices and frequent therapeutic use, were sold by every vending place approached. PZQ, which is more expensive, was not regularly obtainable–while regularly found along the shores of highly infested Lake Victoria, it was only sporadically sold in the West African region of research.

Altogether, 88 different batches of anthelminthic drugs were obtained: 21 were collected in Ghana, 23 in Burkina Faso and Côte d’Ivoire, and 44 in Tanzania. In Ghana, 13 ABZ, 4 MBZ and 1 PZQ product(s) were purchased, in both neighbours, another 9 ABZ, 8 MBZ and again 1 PZQ brand(s). In Tanzania, 15 ABZ (4 of them were veterinarian medicines), 8 MBZ and 5 PZQ brands could be collected. 39 of these 64 products were manufactured in the three Asian countries of China, India and South Korea, of which Indian pharmaceutical companies produced the overall majority (n = 33). African manufacturers from Côte d’Ivoire, Ghana, Kenya, South Africa, Tanzania, and Togo produced another 19, with a majority of them coming from (and being distributed in) Ghana. Only 5 of the anthelminthics were manufactured in Europe, one product came from Mexico (S1 Table).

44 batches of anthelminthic medicines were collected in the northwest of Tanzania, focussing on centres of Lake Zone (including Bukoba, Musoma, Mwanza and Nansio) and Kigoma Region (with Kasulu and Kigoma). Tablets, which had initially been purchased in Mwanza, were directly transferred to Würzburg where they were stored in air-conditioned laboratories of MMI and JMU for further analysis. All other products were brought to BMC Mwanza and stored at the hospital pharmacy at a medium temperature of 21°C. Those were further assayed with the Minilab before shipping them to Germany.

**Fig 1:**
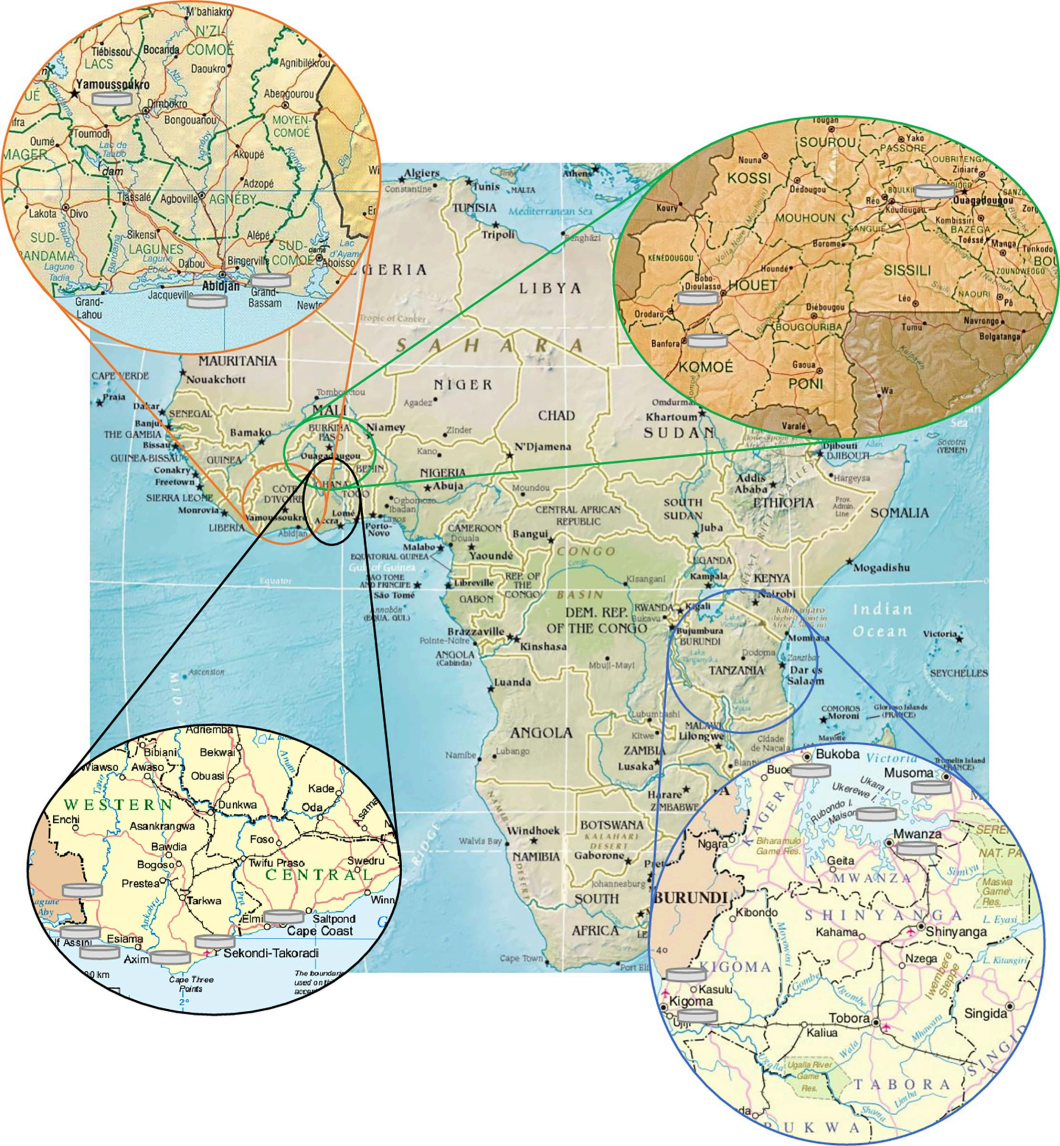
Places of sample collection in Burkina Faso, Côte d’Ivoire, Ghana and Tanzania. A grey tablet does not indicate a certain quantity [28; adapted].

44 different West African batches were obtained in two different styles: sampling in Ghana was concentrated to the rural Western Region around the small village of Eikwe and the cities of Cape Coast and Takoradi. In Burkina Faso, products were acquired in larger cities (Banfora, Bobo Dioulasso and Ouagadougou). The same approach was chosen in Côte d’Ivoire in (Abidjan, Grand Bassam and Yamoussoukro). All West African samples were stored and carried well protected from heat and dust during transfer to Europe (Fig 1).

The vast majority of samples was obtained in official pharmacies. If possible, medicines were also purchased from small simple stalls, selling common ‘important’ medicines (over-the-counter shops in Ghana, *duka la (ma)dawa muhimu* in Tanzania) or, in Burkina Faso, from unofficial street vendors. Transnationally, an overt approach to the different drug-selling facilities was chosen. They were randomly accessed by both local and German colleagues and openly asked for disparate anthelminthic medicines available. Critical questions about the intended purchase were never raised; as this study aimed at analysing local markets and not at exposing suppliers of substandard and / or falsified drugs, there was no need for an invented explanation to blindfold vendors. Consulting the lists of drugs officially licensed by national drug authorities (in Ghana and Tanzania) [29,30] and evaluating the first impressions in Burkinabé and Ivorian pharmacies, attention was given to the wide variety of especially ABZ and MBZ disposable in each country. Whenever retrievable, supposedly non-licensed or loosely sold tablets without primary packaging were included.

### Appearance and packaging

All products were subjected to visual examination according to the WHO ‘Be Aware’ tool, distinguishing between the packages and the tablets themselves (not applicable for the 5 included suspensions) [23]. 3 brands purchased in Tanzania were exclusively sold openly, wherefore only an indicative statement about their packaging could be made from online research; another 3 drugs from Burkina Faso were just sold in blisters, without secondary packaging. According to the brand names from the packages / blisters, 7 products acquired were originator brand drugs (ABZ: *Zentel* from France, India and South Africa; MBZ: *Vermox* from Portugal and South Africa; PZQ: *Biltricide* from Germany and *Cesol* from Mexico). The majority of 48 brands was represented by branded generics: 19 seemed to be registered trademark brands, 1 seemed to a be non-registered trademark brand and 28 did not show any trademark labelling. 9 products were sold as generic drugs under their international non-proprietary names. According to official figures in Ghana and Tanzania, 9 of the 46 products obtained in both countries were not licensed for sale by the time of purchase (*Cesol* is being donated for and applied in preventive chemotherapy of schistosomiasis in Tanzania). Apart from 5 drugs not indicating their date of manufacture, examination of the packages did not expose a medicine openly deviating from the WHO tool (S2 Table). A heterogenous texture (in 7 drugs) and some minor breaks and cracks (in 8 drugs) were the most obvious alterations in the outward appearance of the tablets - none of these deviations though pointed out on a substandard and / or falsified medicine (S3 Table).

### Minilab results

#### Mass uniformity

To pass this criterion, the mass of at least 18 out of 20 samples (respectively 90%, whenever not enough samples had been acquired) should not exceed a limit of ± 5.0% from the calculated average mass, and at most two tablets should not exceed a limit of ± 10.0%. 5 out of 58 tested brands assayed failed to meet specification limits and exceeded the limit of 5.0%. Herein, no product was unveiled to contain samples deviating by more than 10%. Specimen of Ghanaian *Trazole-500* failed in this testing owing to a distinctive variation around the average mass with a relative standard deviation of 6.25%. Only 33.3% of the tablets accorded with specification limits (S4 Table).

#### Disintegration times

Anthelminthics like ABZ or MBZ acting intraluminally against their targets rely on a thorough local release of the API. In disintegration analyses however, 12 out of 56 products assayed (8 ABZ and 4 MBZ brands) completely failed to meet the specification limit of 30 minutes. Of these, only Tanzanian *Alben* disintegrated within extended time of analysis (45 minutes). Thrice, variable disintegration times were obtained within one product: Ghanaian *Wormron 400* presented with 1 batch closely (23 to 32 minutes) and a second fully (38 to 47 minutes) failing; South Korean *Alzental* acquired in Tanzania presented with 1 batch in range (12.5 to 14 minutes) and another 1 completely failing (37.5 to 45 minutes and beyond); 1 out of 3 batches of Tanzanian *Praziquantel 600* failed (25 to 35.5 minutes) while the other 2 were well in range (5.5 to 16 minutes). Overall, 73.2% (n = 41) of anthelminthic brands of tablets gathered passed this Minilab method, varying between almost instant disintegration (*T-Medazol* / MBZ, all within 1 minute) and just passing (*Sequizol* / ABZ, 24 to 29 minutes). 3 medicines obtained in Tanzania (*Zentel* / ABZ, *Ashialben 600* / veterinarian ABZ and *Vermox* / MBZ) were not taken into account due to limited number of samples purchased (S4 Table).

#### Thin-layer chromatography

All 83 disparate batches of anthelminthic tablets obtained in Africa (43 from West Africa, 40 from Tanzania) were subjected to TLC before further evaluating them by HPLC (S4 Table). Each sample and, if possible, each batch likewise were double-checked–the West African products twice in Würzburg, the Tanzanian products acquired in September and October 2018 initially in Mwanza and then re-evaluated in Germany. Tablets passed criteria if the intensity of the principal spots was detected between LSL and USL, and if the retention factor (RF) of the samples corresponded with the RF of the reference substance (deviations of ± 10% included).

In total, no distinctively substandard or falsified medicine was encountered: all samples were confirmed to contain the indicated API. Content of API could merely roughly be estimated, especially when close to the 80% LSL or to the 100% USL (Table 2). 61 of the batches were in range–only 4 batches seemed to just have failed the 80% limit, and 18 batches appeared to have exceeded the 100% limit. Within the latter ones, predominantly MBZ batches were assessed to emit a more intense fluorescence than the USL.

**Table 2:** TLC results.

TLC results	ABZ (n = 45)	MBZ (n = 23)	PZQ (n = 11)	Vet. ABZ (in Tanzania) (n = 4)	Total (n = 83)
In range (API 80%– 100% l.c.)	95.6% (43)	21.7% (5)	100.0% (11)	50.0% (2)	73.5% (61)
API exceeding 100% l.c.	2.2% (1)	73.9% (17)	0.0% (0)	0.0% (0)	21.7% (18)
API undercutting 80% l.c.	2.2% (1)	4.3% (1)	0.0% (0)	50.0% (2)	4.8% (4)
Falsified / counterfeit API	0.0% (0)	0.0% (0)	0.0% (0)	0.0% (0)	0.0% (0)

### High-performance liquid chromatography

In total, 83 batches were tested by HPLC. Depending on availability, 1 to 6 samples per batch (adding up to 182 samples) were examined. Herein, not a single batch was encountered to contain a wrong API or to be of substandard quality, which, by WHO definition, refers to ‘authorized medical products that fail to meet either their quality standards or their specifications, or both’ [31]. Regarding the determination of content, current pharmacopoeiae [25,26] require an amount between 90% and 110% of the declared API content. All of the 10 PZQ batches passed the specification. Only 1 out of 25 MBZ batches did not comply with the specifications, with an average content of 78.7%. 8 out of 48 ABZ batches had an average content between 83.3% and 89.9%, and 1 batch an amount of 74.1% of the declared API content (Tables 3 and S4). Overall, 10 out of 83 batches were underdosed (Table 4).

**Table 3:** HPLC results.

HPLC results	ABZ (n = 48)	MBZ (n = 25)	PZQ (n = 10)	Total (n = 83)
In range (API 90%– 110% l.c.)	81.3% (39)	96.0% (24)	100.0% (10)	88.0% (73)
API exceeding 110% l.c.	0.0% (0)	0.0% (0)	0.0% (0)	0.0% (0)
API undercutting 90% l.c.	18.8% (9)	4.0% (1)	0.0% (0)	12.0% (10)
Falsified / counterfeit API	0.0% (0)	0.0% (0)	0.0% (0)	0.0% (0)

**Table 4:** HPLC results of failed batches.

#	Average content of failed batches [%] (individual samples when applicable)
GH_A2	87.7 (86.8 / 88.6)
GH_A7	87.2 (85.7 / 88.6)
GH_A8	87.3 (86.5 /88.2)
GH_A9	87.7 (72.3 / 75.9)
GH_A12	84.6 (76.6 / 92.7)
BF/CI_A1	84.1 (83.3 / 85.0)
BF/CI_A9 (TE-6717)	86.4 (85.0 / 87.9)
TZ_A4 (ALZET S002)	89.9 (88.7 / 89.8 / 91.2)
TZ_A4 (ALZET S001)	86.3
TZ_M2	76.3 / 81.2

### Dissolution profiles

**Fig 2:**
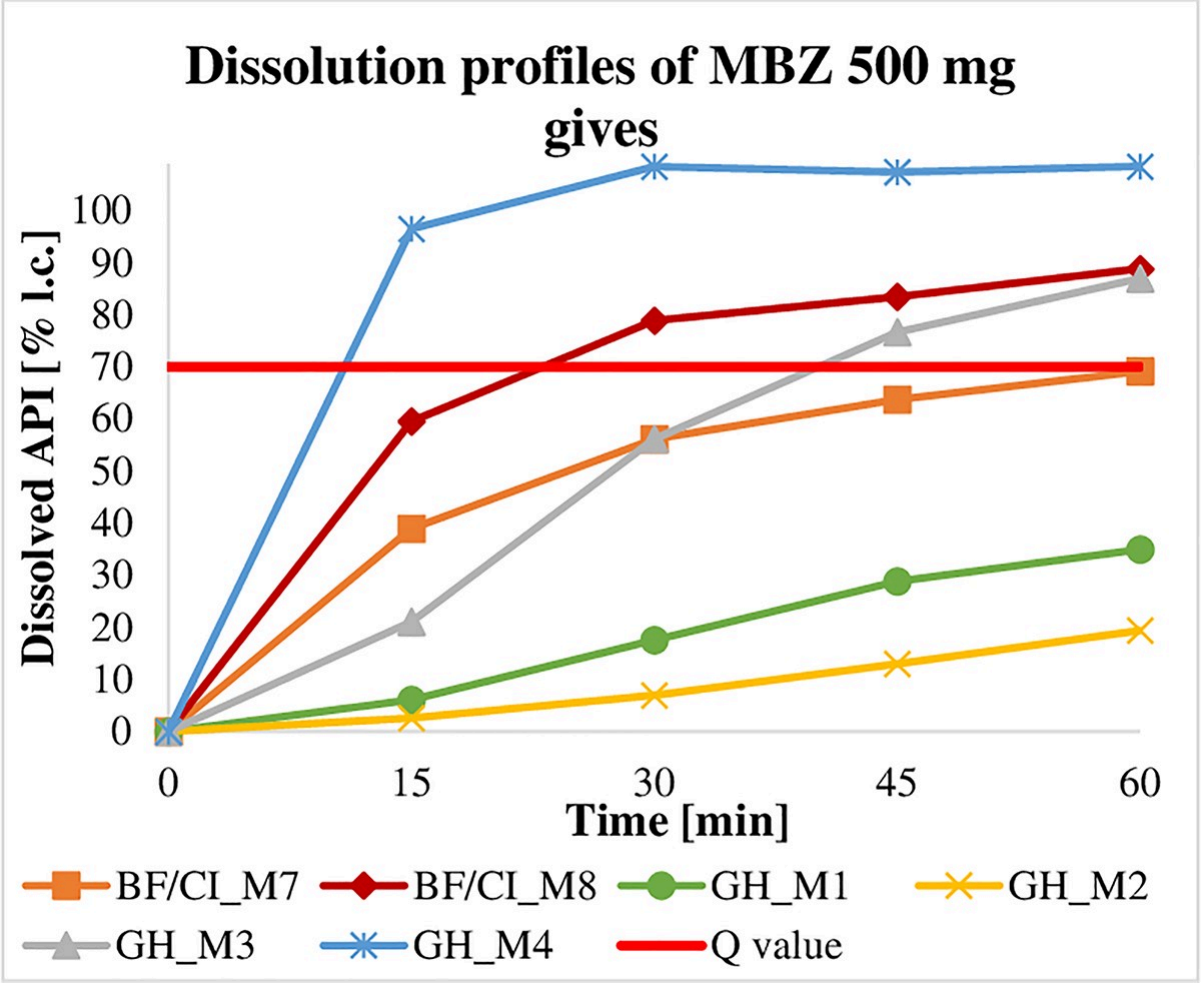
Dissolution profiles of 500 mg MBZ samples (Q = 70%). 100 mg gives are discussed in Chapter 4.2.

**Fig 3:**
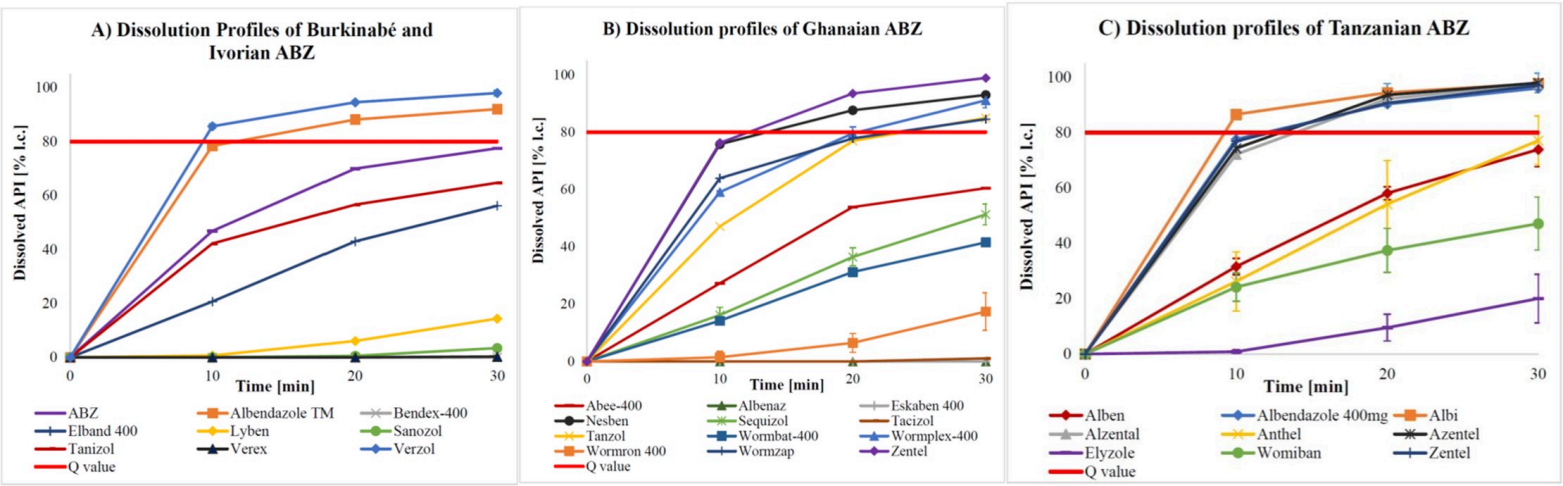
Dissolution profiles of ABZ samples from Burkina Faso and Côte d’Ivoire (A), Ghana (B) and Tanzania (C) (Q = 80%).

**Fig 4:**
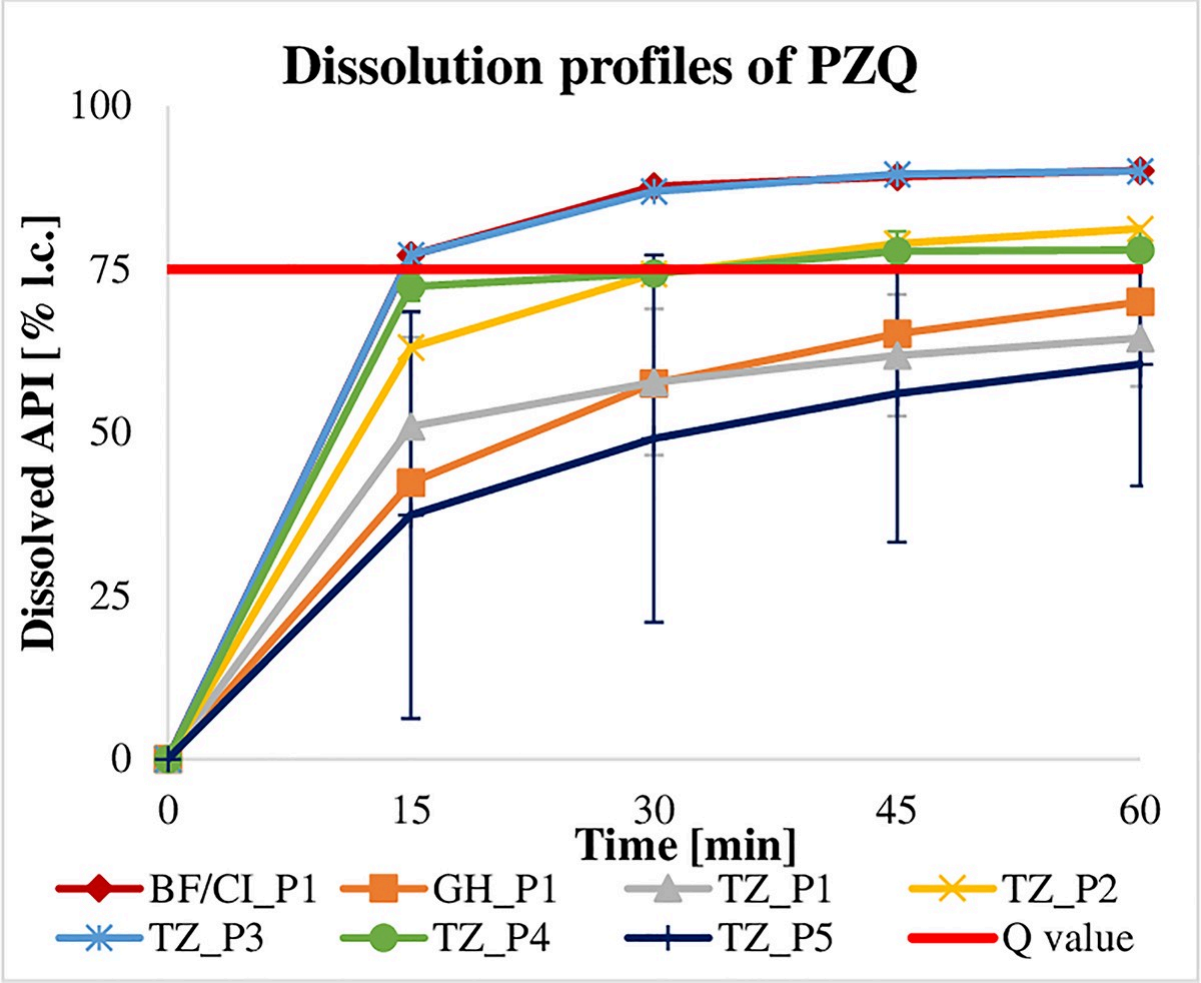
Dissolution profiles of PZQ samples from all four countries (Q = 75%).

Requirements were met if the quantity of API dissolved from a dosage form was equal to or larger than the minimal quantity (Q) listed in the corresponding monographs of current pharmacopoeiae. Q is the specified amount of dissolved API expressed as a percentage of the labelled content [25,27]. Owing to a limited number of samples, the dissolution profile of just one tablet per batch could only be evaluated. According to USP-NF 41 [27] and Ph. Int. 7 [25], a dissolution profile is defined by a testing of at least 6 tablets over 3 levels, which was not reached and thus limited the expressiveness of the generated data. Nevertheless, a majority of dosage forms revealed to show deficits in their dissolution (Table 5). Here, MBZ brands have to be highlighted as none of the 100 mg formulations (n = 14)–applying the method described in Ph. Int. 7 [25] (0.1 M HCl as dissolution medium for 100 mg formulations and 0.01 M HCl + 1% SDS as dissolution medium for 500 mg formulations)–reached the pre-defined Q value (60%), regardless of whether the tablet had disintegrated or not. To double-check these findings, available samples were additionally tested according to the USP-NF 41 method [27], which does not differentiate between 100 mg and 500 mg formulations and runs with 0.1 M HCl + 1% SDS as dissolution medium in both cases. In accordance with USP-NF 41, 5 out of 8 batches (62.5%) passed the specification (Q = 75%). Under the same testing conditions (except for sampling time and dilution), 50.0% (n = 3) of the 500 mg MBZ tablets released more API than the limit of Q = 70%, which is required by the Ph. Int. 7 [25], while the remaining 50% failed to do so (Fig 2). ABZ (Fig 3) and PZQ (Fig 4) did not perform sufficiently either: only 41.9% (n = 18) of the ABZ samples (Q = 80%) and 50% (n = 5) of the PZQ samples (Q = 75%) assayed passed specifications (S4 Table).

**Table 5:** Dissolution results.

Dissolution results	ABZ (n = 43)	MBZ 100 mg (n = 14 */8 **)	MBZ 500 mg (n = 6)	PZQ (n = 10)	Total (n = 73 */67 **)
Value ≥ Q	41.9% (18)	0.0% (0) * 62.5% (5) **	50.0% (3)	50.0% (5)	35.6% (26) * 46.3% (31) **
Value < Q	58.1% (25)	100.0% (14) * 37.5% (3) **	50.0% (3)	50.0% (5)	64.4% (47) * 53.7% (36) **

* MBZ 100 mg tested with method described in Ph. Int. 7

** MBZ 100 mg tested with method described in USP-NF 41

## Discussion

In an ambitious and successful combat against NTDs, warranting sufficient access to quality-assured therapeutics must not be neglected. By this study, we expanded the African map of reproducibly assessed Albendazole, Mebendazole and Praziquantel by East African and West African products. Herein, some medicines are not exclusively available in the countries of collection but also - as seen in West Africa - in neighbouring ones: products found in Tanzania tend to be officially licensed throughout the East African Union, Malawi or Zambia; Ghanaian anthelminthics may be sold eastward towards Nigeria as well; and the drugs collected from the two francophone countries are likely to be distributed in surrounding francophone nations. Owing to the abundance of generics encountered on the local markets, an exhaustive evaluation was not aspired but rather a realistic cross-sectional assay of samples sold and purchased in the respective environment.

**Fig 5:**
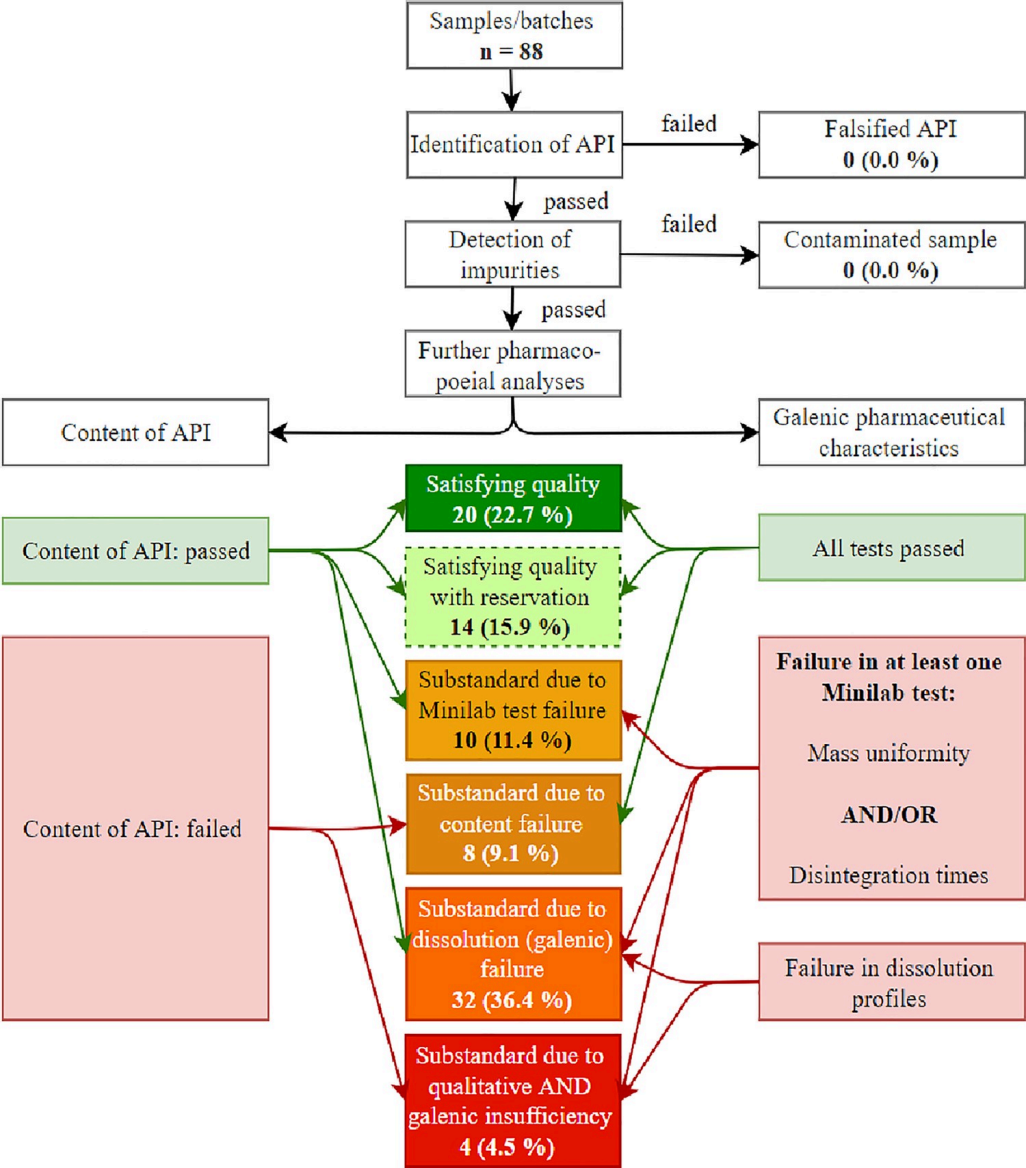
Flow chart of categorization of the African samples obtained. Due to its likelihood in our results, galenic insufficiency was evaluated to be more important than insufficiency of content. Dissolution profiles were regarded as more expressive than Minilab results; failure of dissolution and disintegration regularly coincided. HPLC results surpassed TLC results as the latter ones are not quantitative. Satisfying quality with reservation: not all tests could be performed; the ones performed resulted in acceptable quality.

A satisfying quality could merely be recognised in 22.7% of the disparate batches gathered. Conversely, 61.4% of them failed in at least one category (content by HPLC and TLC, dissolution profiles, Minilab working with mass uniformity and disintegration)– 4.5% (4 / 88) even in both HPLC and dissolution (Fig 5). Even though not regularly reported accordingly, galenic insufficiency exceeded the issue of diminished content.

### Active pharmaceutical ingredient as most critical feature of anthelminthics?

In previous studies on QA of anthelminthic drugs, the question for falsification of locally available samples was regularly determined to be the principal goal of research. According to the WHO, falsified drugs are defined as ‘medical products that deliberately / fraudulently misrepresent their identity, composition or source’ [31]. Herein, particularly anti-infective medicines seem to be prone to alterations: from 2013 to the first months of 2020, WHO drug alerts on 31 anti-malarial batches (Chloroquine, Quinine, Sulfadoxine/Pyrimethamine and Artemether/Lumefantrine) as well as on eight antibiotic batches (Amoxicillin mostly in combination with Clavulanic Acid, Penicillin V and Cefixime) were issued, most of them detected in Africa [32]. Such SF medicines are known to induce growing antimicrobial resistances, which nowadays regularly occur in antibiotics and, increasingly, in anti-malarials [33–35]. Although complete absence of stated API in anthelminthic medicines has virtually not been reported [16–22], incentives for this are obvious: about 612 million of African people were infected with at least one NTD in 2018 - they were approached by both therapy and of internationally donated preventive chemotherapy, which reached approximately 60% of those who were in need of it [36]. Despite not having to fear them on a large scale, resistant parasites have already been described [37]. However, as patients suffering from schistosomiasis or STH (especially people at risk like school-aged children who are regularly treated by MDA) may not present with clinically striking and characteristic symptoms, they will not primarily opt for ABZ, MBZ or PZQ. Instead, they seem to rather ask for medicines, which are more realistically expected to be of help: anti-infective drugs like antibiotics and anti-malarials, analgesics or vitamins [38]. This tendency sustains a certain neglect of QA in deworming drugs. To emphasise the necessity of quality-assured anthelminthics, we aimed at evaluating their content in the four countries of research despite being aware of earlier inobtrusive findings. As to be expected, sheer presence of API in Burkinabé, Ghanaian, Ivorian and Tanzanian anthelminthics did not seem to be the most vulnerable aspect of QA - not in a single drug we discovered a complete lack of or a wrong API.

The content of API was assessed by two different approaches: the GPHF Minilab, a screening method whose suitability in low- and middle-income countries is acknowledged but whose semi-quantitative results are recommended to be assessed only in combination with other (screening or confirming) methods [39,40]. A confirming method, the HPLC, was then applied in almost all samples collected to quantify the amount of API. In this study, the gold standard method HPLC was able to illustrate the applicability of the screening tool in terms of categorizing the amount of API in anthelminthic medicines. The combined results showed only minor deviations from the ranges of acceptance: screening deworming drugs, predominantly MBZ products appeared to exceed specification limits; confirming them, 10 batches missed these limits, ranging from averages of 74.1% to 89.9%. Our results accorded with comparable studies on deworming drugs for TLC [22] and HPLC [16–21], even though MBZ brands were analysed to be concentrated more irregularly [17,18].

Since TLC and HPLC set ranges of acceptance that differ by 10% (TLC: 80%– 100% l.c.; HPLC: 90%– 110% l.c.), the results drawn from our research must be evaluated at a broader view. In TLC, samples that are close to specification limits can hardly be assessed as ‘passing’ or ‘failing’; it is a subjective evaluation based on nuances that cannot deliver a reliable classification. On the other hand, products that are tested by HPLC and that just undercut the 90% limit would not be identified as such by the Minilab. Nevertheless, an overall concordance can definitely be acknowledged–despite the observation that both likelihood and concentration of excessive API appear to be over-estimated in MBZ samples by the newly added Minilab method of applying methanolic sulphuric acid and subsequent 366 nm UV light to duly prepared TLC plates. In all but 2 batches, the subjective assessment of ABZ, MBZ and PZQ by TLC (taking the uncertainties around specification limits into account) was confirmed by HPLC. Conflicting results were only seen in *De Wome 500* and *Mebendazole BP 500 mg*. According to TLC results, *De Wome 500* tended to contain less than 80% l.c. whereas HPLC analyses came up with an amount of 101.2%. With *Mebendazole BP 500 mg*, it was the other way around: TLC results showed a content of beyond 100% l.c. while the amount determined by HPLC was set at an average of 78.8% (S4 Table). These differing findings may be explained by the small number of samples analysed. Not enough tablets were purchased to increase the power of the results drawn or to depict a possible heterogeneity within these two batches. Moreover, due to the subjective evaluation of TLC, a difference of 5–10% l.c. may easily be considered to emit a very similar fluorescence.

### Fluctuating results in testing for galenic features

Even though researchers had used to define the content of API as most relevant aspect in QA [18,41], duly performed assays on deworming agents identified significant uncertainties in the release of an API from its pharmaceutical formulation [16–18,22]. These results were confirmed and complemented by our findings: just more than one fourth (26.8%) of the obtained brands of oral solid forms included at least one batch failing to disintegrate within 30 minutes.

By the two methods adopted, both the solidness of tablets analysed and the aqueous solubility into the bloodstream could be determined. This is particularly important as, according to the Biopharmaceutics Classification System, ABZ is listed in class IV, PZQ in class II and MBZ in both–which means that the solubility of all three anthelminthics must be regarded as ‘low’. Additionally, class IV medicines like ABZ are poorly absorbed in the small intestine [42]. Consequently, for ABZ and MBZ products aiming at intestinal helminths it is important to fully disintegrate in order to successfully compete for the target protein, a β-subunit of the nematodal tubulin molecule [43]. To reach both intraluminal and extraintestinal helminths, ABZ (for reasons of activation into its therapeutically relevant sulphoxide), MBZ and PZQ need to dissolve as thoroughly as possible to have enough API overcome hepatic first pass metabolism. Whenever tablets do not disintegrate nor dissolve appropriately, there is a risk of insufficient therapy of systemic helminthiases inducing resistances - analogous to the aspects mentioned above: not enough API may reach and harm the helminths. Belew et al. illustrated that deficiencies of ABZ samples in dissolution profiles detected in vitro do correlate with diminished curing and egg reduction rates in patients [16]. They compared the two ABZ products *Bendex* (also included in our study) and *Ovis* regarding their (physico-)chemical quality and their efficacy against STH. While patients with ascariasis and trichuriasis experienced no differing efficacy of treatment, *Bendex* was significantly less effective in ancylostomiasis than *Ovis*. This result was reflected in a significantly slower dissolution rate of *Bendex*.

Disintegration had not been described as quality prone to deficiencies. Rarely assayed in deworming medicines so far, disintegration times seemed to routinely comply with respective criteria [17,18,22]. In contrast to these previous findings, 10 products from the four countries of research were encountered to not disintegrate at all. Even though all of them were labelled as chewable tablets, this identification does not exclude them from pharmacopoeial criteria. Despite differentiating between ABZ / MBZ chewable and non-chewable formulations, Ph. Int. 7 does not provide disparate methods of analysing disintegration times. Both monographs moreover state that chewable tablets ‘may be chewed, swallowed whole or crushed and mixed with food or liquid’–implying that galenic characteristics should not depend on sufficient fragmentation of the tablets prior to gastric passage [25].

Insufficient dissolution profiles are reported for all three APIs [16–18,22]; some of the brands tested in vitro in this research were analysed to fail in previous studies as well: *Natoa* / MBZ from Kenya (Q = 7.9% [22] compared to no more than Q = 1.4%) dissolved just marginally. *Bendex* mentioned above (Q = 20.1% [16] compared to non-dissolution) and *Bermoxel* / PZQ from Cyprus (Q = 63.4% [17] compared to no more than Q = 69.7%) were confirmed to fail, too. In contrast to the occasionally or moderately failing dissolution profiles reported, our results illustrated a larger and wider spectrum: 53.7% (n = 36, using the USP-NF 41 method for 100 mg MBZ formulations) of all tested batches (n = 67) of solid formulations did not meet the respective criteria - of these, 8 samples (7 ABZ batches, 1 MBZ batch) did not even dissolve 10.0% of the API. Overall, ABZ samples showed the worst results. 25 (58.1%) out of 43 samples did not reach the required Q value. In contrast, 42.9% (n = 6) of the MBZ batches and 50.0% (n = 3) of the PZQ batches failed the specification.

**Fig 6:**
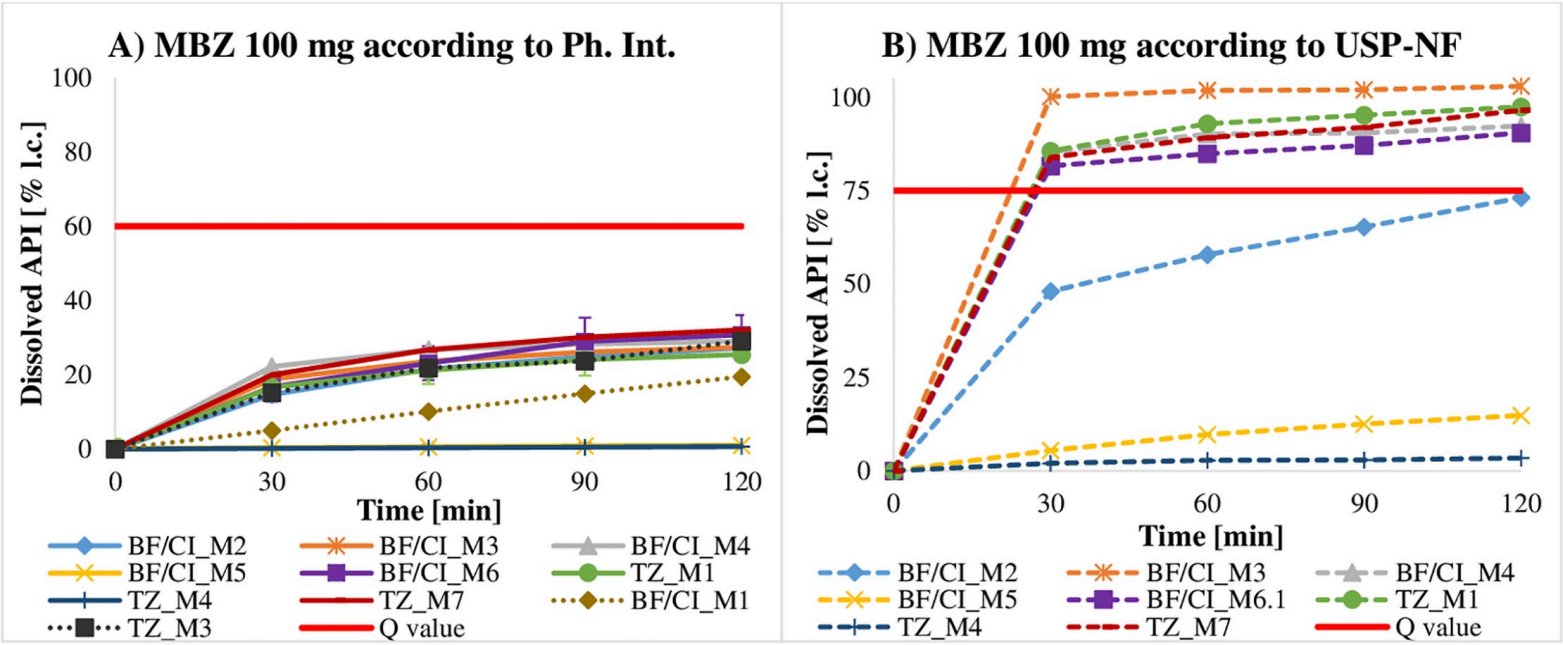
**Dissolution profiles of 100 mg gives of MBZ according to Ph. Int. 7 (A) and USP-NF 41 (B).** Colours in both figures correspond to a respective product. Dotted graphs in A): samples were merely analysed referring to Ph. Int. 7.

Remarkably, dissolution results of 100 mg MBZ formulations differed strongly depending on the method described in either Ph. Int. 7 (Fig 6A) or USP-NF 41 (Fig 6B). This disparity between both methods may be seen in the composition of the dissolution medium. While Ph. Int. 7 applies 0.1 M HCl, USP-NF 41 recommends a solution of 1% SDS in 0.1 M HCl. Working with purely 0.1 M HCl, none of the tested tablets reached the required Q value - whereas dissolving 100 mg MBZ gives in 0.1 M HCl + 1% SDS enables 5 out of 8 tablets to pass the specification. This observation is confirmed by Garbuio et al. [44] who examined the dependence of the solubility of MBZ on the concentration of the added SDS. From 0.1 M HCl without added SDS to a concentration of 1.5% SDS, the amount of released API increased. During the initial methodical evaluation, samples of *Vermox* 100 mg from Germany were tested applying both dissolution methods, and both specifications led to satisfactory results. This formulation though contained surfactant SDS–which reaffirmed the statement that, even in small quantities, SDS significantly increases the solubility of MBZ. Therefore, one may assume that the tablets purchased in Tanzania and West Africa did not contain any SDS in their formulation. This observation has not been confirmed nor refuted so far, since no information on the ingredients of the evaluated samples has yet been obtained. Overall, the method described in Ph. Int. 7 appears to be not suitable for 100 mg MBZ formulations without added SDS.

## Conclusion

Anthelminthic medicines available in Burkina Faso, Côte d’Ivoire, Ghana and Tanzania were not encountered to be falsified. In most products, enough content was detected to comply with international pharmacopoeiae. Analysing the amount of API, TLC as key feature of the GPHF Minilab - even though limited in its expressiveness - delivered reliable screening results, which were sufficiently confirmed by HPLC. Galenic characteristics of the brands assayed however were determined to be more uncertain and variable than expected. Disintegration and particularly dissolution results raised suspicion of impaired bioavailability in more than 50% of the products. As a reduced in vitro performance of anthelminthics in galenic testing was shown to correlate with a diminished in vivo response in patients [16], our findings induce alarming consequences not only for the therapy but also for the prevention–considering the application and outcomes in mass drug administration–of helminthiases. To thus guarantee a secure access to good-quality medicines, we advocate regular, perhaps intensified QA on anthelminthic medicines available on local markets and can only emphasize the recommendation for the use of QA generic products (by prequalification through the WHO or Stringent Regulatory Authorities) in MDA. A comprehensive database of anthelminthic quality and composition in low- and middle-income countries contributes to the international efforts against NTDs by quality secured therapeutics–the target of the WHO NTD Roadmap to finally terminate both schistosomiasis and STH as public health burden [8] will become just more attainable.

## Ethics

Research Clearance Certificate Number CREC/304/2018 from CUHAS Mwanza. No ethical clearance required at JMU Würzburg.

## Supporting information

S1 Table Details of sample collection of Albendazole, veterinarian Albendazole, Mebendazole and Praziquantel. (DOCX)

S2 Table Appearances of the packaging of Albendazole, veterinarian Albendazole, Mebendazole and Praziquantel. (DOCX)

S3 Table Visual examination of Albendazole, veterinarian Albendazole, Mebendazole and Praziquantel formulations. (DOCX)

S4 Table Pharmacopoeial analysis of Albendazole, veterinarian Albendazole, Mebendazole and Praziquantel. (DOCX)
