# Supplementary material for: Quality and composition of Albendazole, Mebendazole and Praziquantel available in Burkina Faso, Côte d’Ivoire, Ghana and Tanzania
Source: PLoS Negl Trop Dis. 2021 Jan 25;15(1):e0009038. doi: 10.1371/journal.pntd.0009038 (PMC7861518; doi:10.1371/journal.pntd.0009038)
Supplement: S1 Table — (DOCX) [file pntd.0009038.s001.docx]

**S1 Table: Details of sample collection of Albendazole, veterinarian Albendazole, Mebendazole and Praziquantel** (**bold**: did not pass specifications)

| # | Brand name (batch number) | Labelled API content [mg] | Country of origin | Town of collection | Selling agent / facility [Hospital pharmacy; Pharmacy; Dispensary; over-the-counter (OTC); Duka la (ma)dawa (muhimu); Street vendor] | Date of expiry | Officially licensed for treatment in the respective country by the time of purchase (only applicable in Ghana and Tanzania) [yes / no] |
| --- | --- | --- | --- | --- | --- | --- | --- |
| GH_A1 | Abee-400 | 400 | India | Eikwe | OTC | Feb 21 | **no** |
| GH_A2 | Albenaz | 400 | Ghana | Elubo | OTC | Jan 22 | yes |
| GH_A3 | Albendazole oral suspension | 400/20 mL | India | Takoradi | Pharmacy | Jun 20 | **no** |
| GH_A4 | Eskaben 400 | 400 | Ghana | Eikwe | OTC | Dec 21 | yes |
| GH_A5 | Nesben | 200 | Ghana | Takoradi | Pharmacy | Oct 22 | yes |
| GH_A6 | Sequizol (AT97) | 400 | India | Cape Coast | Pharmacy | Jun 20 | yes |
|  | Sequizol (AT01) |  |  |  | Pharmacy | Jan 21 |  |
| GH_A7 | Tacizol | 400 | India | Cape Coast | Pharmacy | Jan 21 | yes |
| GH_A8 | Tanzol | 400 | India | Essiama / Takoradi | Pharmacy | Jun 21 | yes |
| GH_A9 | Wormbat-400 | 400 | Ghana | Axim | Pharmacy | Sep 20 | yes |
| GH_A10 | Wormplex 400 (WPTP0038) | 400 | India | Eikwe | Pharmacy | Jun 22 | yes |
|  | Wormplex 400 (WPTP0039) |  |  | Takoradi | Pharmacy | Jul 22 |  |
| GH_A11 | Wormron 400 (WA1806) | 400 | India | Eikwe | OTC | Jun 21 | **no** |
|  | Wormron 400 (WA1801) |  |  |  | *Prov. by St. Martin de Porres* | Jan 21 |  |
| GH_A12 | Wormzap | 400 | Ghana | Cape Coast | Pharmacy | Jul 21 | **no** |
| GH_A13 | Zentel | 200 | India | Axim | Pharmacy | Jan 23 | yes |
| BF/CI_A1 | ABZ | 400 | India | Bobo Dioulasso / Ouagadougou | Pharmacy | Dec 20 | N/A |
| BF/CI_A2 | Albendazole TM | 400 | Togo | Grand Bassam / Ouagadougou | Pharmacy | Nov 20 | N/A |
| BF/CI_A3 | Bendex-400 | 400 | India | Yamoussoukro | Pharmacy | Dec19 | N/A |
| BF/CI_A4 | Elband 400 | 400 | India | Banfora | Pharmacy | Apr 21 | N/A |
| BF/CI_A5 | Lyben | 400 | China | Abidjan | Pharmacy | Sep 21 | N/A |
| BF/CI_A6 | Sanozol | 400 | India | Yamoussoukro | Pharmacy | Oct 20 | N/A |
| BF/CI_A7 | Tanizol | 400 | India | Banfora | Street vendor | Jul 21 | N/A |
| BF/CI_A8 | Verex (186) | 400 | Côte d'Ivoire | Abidjan | Pharmacy | Oct 21 | N/A |
|  | Verex (175) |  |  | Ouagadougou | Pharmacy | Mar 21 |  |
| BF/CI_A9 | Verzol (TE-6717) | 400 | India | Grand Bassam | Pharmacy | Feb 21 | N/A |
|  | Verzol (TE-6677) |  |  | Grand Bassam | Pharmacy | Jan 21 |  |
|  | Verzol (TE-6716) |  |  | Ouagadougou | Pharmacy | Feb 21 |  |
| TZ_A1 | Alben (180018) | 200 | Tanzania | Bukoba | Pharmacy | Jun 22 | yes |
|  | Alben (170023) |  |  | Mwanza | Hospital pharmacy | Mar 21 |  |
|  | Alben (180012) |  |  | Nansio | Duka la dawa | Jun 22 |  |
| TZ_A2 | Albendazole 400mg | 400 | India | Nansio | Dispensary | N/A | **no** |
| TZ_A3 | Albi | 400 | South Korea | Mwanza | Pharmacy | Nov 19 | yes |
| TZ_A4 | Alzental (ALZET S002) | 400 | South Korea | Mwanza | Pharmacy | Sep 20 | yes |
|  | Alzental (ALZET S001) |  |  | Mwanza | Pharmacy | May 20 |  |
| TZ_A5 | Anthel (BV6006) | 400 | India | Musoma | Duka la dawa | Mar 19 | yes |
|  | Anthel (BV7004) |  |  | Mwanza | Pharmacy | Feb 20 |  |
| TZ_A6 | Azentel (360766) | 400 | India | Kigoma | Pharmacy | Sep 20 | yes |
|  | Azentel (350579) |  |  | Musoma | Pharmacy | Jun 19 |  |
|  | Azentel (370425) |  |  | Mwanza | Pharmacy | Apr 21 |  |
| TZ_A7 | Benpham suspension | 400/10 mL | India | Bukoba | Pharmacy | Apr 21 | yes |
| TZ_A8 | Elyzole (5I07) | 200 | Kenya | Mwanza | *Prov. by CUHAS* | Aug 19 | yes |
|  | Elyzole (7G131) |  |  | Kasulu | Pharmacy | Jun 21 |  |
|  | Elyzole (6K01) |  |  | Kigoma | Duka la dawa | Oct 20 |  |
| TZ_A9 | Womiban (FWX1706) | 400 | India | Kasulu | Pharmacy | Sep 21 | yes |
|  | Womiban (FWX1701) |  |  | Mwanza | Pharmacy | Dec 20 |  |
| TZ_A10 | Zentel | 400 | South Africa | Mwanza | Hospital pharmacy | Jul 20 | yes |
| TZ_A11 | Zentel suspension | 400/20 mL | France | Mwanza | Pharmacy | Jan 20 | yes |

| # | Brand name (batch numbers) | Labelled API content [mg] | Country of origin | Town of collection | Selling agent / facility [Hospital pharmacy; Pharmacy; Dispensary; over-the-counter (OTC); Duka la (ma)dawa (muhimu); Street vendor] | Date of expiry | Officially licensed for treatment in the respective country by the time of purchase (only applicable in Ghana and Tanzania) [yes / no] |
| --- | --- | --- | --- | --- | --- | --- | --- |
| vetA1 | Albendafarm 1500 | 1500 | China | Mwanza | veterinarian pharmacy | Jul 20 | yes |
| vetA2 | Albendafarm 2500 | 2500 | China | Mwanza | veterinarian pharmacy | Nov 20 | yes |
| vetA3 | Ashialben 300 | 300 | India | Mwanza | veterinarian pharmacy | Jan 22 | yes |
| vetA4 | Ashialben 600 | 600 | India | Mwanza | veterinarian pharmacy | Feb 20 | yes |

| # | Brand name (batch number) | Labelled API content [mg] | Country of origin | Town of collection | Selling agent / facility [Hospital pharmacy; Pharmacy; Dispensary; over-the-counter (OTC); Duka la (ma)dawa (muhimu); Street vendor] | Date of expiry | Officially licensed for treatment in the respective country by the time of purchase (only applicable in Ghana and Tanzania) [yes / no] |
| --- | --- | --- | --- | --- | --- | --- | --- |
| GH_M1 | De Wome 500 | 500 | Ghana | Takoradi | Pharmacy | Aug 22 | yes |
| GH_M2 | Mentel | 500 | Ghana | Eikwe | *Donation* | Aug 20 | yes |
| GH_M3 | Trazole-500 | 500 | Ghana | Elubo / Essiama | OTC (Elubo) / Pharmacy (Essiama) | May 22 | yes |
| GH_M4 | Vermox | 500 | Portugal | Eikwe | Hospital pharmacy | Jun 20 | yes |
| BF/CI_M1 | Carben | 100 | India | Abidjan | Pharmacy | Dec 20 | N/A |
| BF/CI_M2 | Mébendazole (G07002) | 100 | India | Ouagadougou | Pharmacy | Apr 20 | N/A |
| BF/CI_M3 | Mebendazole (F0164) | 100 | India | Banfora | Pharmacy | Mar 21 | N/A |
| BF/CI_M4 | Mebendazole (MZ-1811) | 100 | India | Banfora | Street vendor | May 21 | N/A |
| BF/CI_M5 | Nebenda | 100 | India | Bobo Dioulasso | Street vendor | Jul 21 | N/A |
| BF/CI_M6 | Oziben (DOR 1702) | 100 | India | Abidjan | Pharmacy | Nov 20 | N/A |
|  | Oziben (DOR 1701) |  |  | Yamoussoukro | Pharmacy | Apr 20 |  |
| BF/CI_M7 | T-Medazol | 500 | India | Abidjan | Pharmacy | Apr 20 | N/A |
| BF/CI_M8 | Wormin 500 (R70E8001) | 500 | India | Grand Bassam | Pharmacy | Dec 21 | N/A |
|  | Wormin 500 (R70E8003) |  |  | Abidjan | Pharmacy | Dec 21 |  |
| TZ_M1 | Astazole (254) | 100 | India | Bukoba | Duka la dawa | Aug 20 | yes |
|  | Astazole (257) |  |  | Musoma | Pharmacy | Dec 20 |  |
|  | Astazole (256) |  |  | Mwanza | (Hospital) Pharmacy | Nov 20 |  |
| TZ_M2 | Mebendazole BP 500mg | 500 | Cyprus | Kasulu (Kabanga) | Duka la dawa | N/A | **no** |
| TZ_M3 | Mebrone-100 | 100 | India | Kasulu | Pharmacy | May 20 | **no** |
| TZ_M4 | Natoa (71356) | 100 | Kenya | Kigoma | Pharmacy | Mar 21 | yes |
|  | Natoa (69900) |  |  | Mwanza | Pharmacy | Aug 20 |  |
| TZ_M5 | Natoa suspension | 600/30 mL | Kenya | Musoma | Pharmacy | Mar 21 | yes |
| TZ_M6 | Vermox | 500 | South Africa | Mwanza | Pharmacy | Apr 21 | yes |
| TZ_M7 | Wormnil | 100 | India | Mwanza | Pharmacy | Feb 21 | **no** |
| TZ_M8 | Wormol suspension | 600/30 mL | Kenya | Musoma | Pharmacy | Apr 20 | yes |

| # | Brand name (batch number) | Labelled API content [mg] | Country of origin | Town of collection | Selling agent / facility [Hospital pharmacy; Pharmacy; Dispensary; over-the-counter (OTC); Duka la (ma)dawa (muhimu); Street vendor] | Date of expiry | Officially licensed for treatment in the respective country by the time of purchase (only applicable in Ghana and Tanzania) [yes / no] | |
| --- | --- | --- | --- | --- | --- | --- | --- | --- |
| GH_P1 | Praziquantel 600 | 600 | Ghana | Eikwe / Cape Coast | Pharmacy | Sep 20 | **no** |  |
| BF/CI_P1 | Biltricide | 600 | Germany | Grand Bassam | Pharmacy | Oct 20 | N/A |  |
| TZ_P1 | Bermoxel (A8AO3) | 600 | Cyprus | Bukoba | Pharmacy | Jan 19 | yes |  |
|  | Bermoxel (N/A) |  |  | Mwanza | Pharmacy | N/A |  |  |
| TZ_P2 | Cesol | 600 | Mexico | Mwanza | *Prov. by CUHAS* | Sep 19 | *Prov. by CUHAS* |  |
| TZ_P3 | Distocide | 600 | South Korea | Mwanza | Pharmacy | Nov 21 | yes |  |
| TZ_P4 | Prazikant (BZ7034) | 600 | India | Kigoma | Pharmacy | Jan 20 | yes |  |
|  | Prazikant (N/A) |  |  | Mwanza | Pharmacy | N/A |  |  |
| TZ_P5 | Praziquantel-600 (140007) | 600 | Tanzania | Bukoba | Dispensary / Pharmacy | Oct 18 | yes |  |
|  | Praziquantel-600 (160004) |  |  | Kasulu | Pharmacy | Jul 20 |  |  |
|  | Praziquantel-600 (170003) |  |  | Mwanza | Pharmacy | Jul 21 |  |  |
