# Supplementary material for: Quality and composition of Albendazole, Mebendazole and Praziquantel available in Burkina Faso, Côte d’Ivoire, Ghana and Tanzania
Source: PLoS Negl Trop Dis. 2021 Jan 25;15(1):e0009038. doi: 10.1371/journal.pntd.0009038 (PMC7861518; doi:10.1371/journal.pntd.0009038)
Supplement: S2 Table — (DOCX) [file pntd.0009038.s002.docx]

**S2 Table:** **Visual inspection of the packaging of Albendazole, veterinarian Albendazole, Mebendazole and Praziquantel** (**bold**: did not pass specifications)

| # | Container and  Closure (yes / no) | Trade / Brand name (* no ®, ** TM  instead of ®) | Name of active  ingredient | Manufacturer's name and logo (* no logo) | Manufacturer's full address | Medicine strength (mg/unit) | Dosage form | Number of units per container | Dosage statement | Batch / lot number | Date of manufacture and expiry (* no date of manufacture) | Storage information | Leaflet or package insert |
| --- | --- | --- | --- | --- | --- | --- | --- | --- | --- | --- | --- | --- | --- |
| GH_A1 | yes | yes* | yes | **no*** | yes | yes | yes | yes | yes | yes | yes | yes | yes |
| GH_A2 | yes | yes | yes | yes | yes | yes | yes | yes | yes | yes | yes | yes | yes |
| GH_A3 | yes | **no** | yes | **no*** | yes | yes | yes | N/A | yes | yes | yes | yes | yes |
| GH_A4 | yes | yes* | yes | **no*** | yes | yes | yes | yes | yes | yes | yes | yes | **no** |
| GH_A5 | yes | yes | yes | yes | yes | yes | yes | yes | yes | yes | yes | yes | yes |
| GH_A6 | yes | yes* | yes | **no*** | yes | yes | yes | yes | yes | yes | yes | yes | yes |
| GH_A7 | yes | yes* | yes | yes | yes | yes | yes | yes | yes | yes | yes | yes | yes |
| GH_A8 | yes | yes** | yes | yes | yes | yes | yes | yes | yes | yes | yes | yes | yes |
| GH_A9 | yes | yes * | yes | yes | yes | yes | yes | yes | yes | yes | yes | yes | yes |
| GH_A10 | yes | yes* | yes | **no*** | yes | yes | yes | yes | yes | yes | yes | yes | yes |
| GH_A11 | yes | yes** | yes | yes | yes | yes | yes | yes | yes | yes | yes | yes | yes |
| GH_A12 | yes | yes* | yes | yes | yes | yes | yes | yes | yes | yes | yes | yes | yes |
| GH_A13 | yes | yes** | yes | yes | yes | yes | yes | yes | yes | yes | yes | yes | yes |
| BF/CI_A1 | yes | yes * | yes | yes | yes | yes | yes | yes | yes | yes | yes | yes | yes |
| BF/CI_A2 | yes | **(no)** | yes | yes | yes | yes | yes | yes | yes | yes | yes | yes | yes |
| BF/CI_A3 | yes | yes* | yes | **no*** | yes | yes | yes | yes | yes | yes | yes | yes | yes |
| BF/CI_A4 | N/A | yes* | yes | yes | yes | yes | yes | N/A | N/A | yes | yes | N/A | N/A |
| BF/CI_A5 | yes | yes | yes | yes | yes | yes | yes | yes | yes | yes | yes | yes | yes |
| BF/CI_A6 | yes | yes* | yes | **no** | yes | yes | yes | yes | yes | yes | yes | yes | yes |
| BF/CI_A7 | yes | yes* | yes | yes | **no** | yes | yes | yes | yes | yes | yes | yes | yes |
| BF/CI_A8 | yes | yes | yes | yes | yes | yes | yes | yes | yes | yes | yes | yes | yes |
| BF/CI_A9 | yes | yes | yes | yes | yes | yes | yes | yes | yes | yes | yes | yes | yes |
| TZ_A1 | yes | yes | yes | yes | yes | yes | yes | yes | yes | yes | yes | yes | yes |
| TZ_A2 | yes | **no** | yes | yes | N/A | yes | yes | yes | yes | N/A | N/A | yes | N/A |
| TZ_A3 | yes | yes* | yes | yes | yes | yes | yes | yes | yes | yes | yes | yes | yes |
| TZ_A4 | yes | yes | yes | yes | yes | yes | yes | yes | yes | yes | yes | yes | yes |
| TZ_A5 | yes | yes* | yes | yes | yes | yes | yes | yes | yes | yes | yes | yes | yes |
| TZ_A6 | yes | yes** | yes | yes | yes | yes | yes | yes | yes | yes | yes | yes | yes |
| TZ_A7 | yes | yes* | yes | yes | yes | yes | yes | yes | yes | yes | yes | yes | yes |
| TZ_A8 | yes | yes* | yes | yes | yes | yes | yes | yes | yes | yes | yes | yes | yes |
| TZ_A9 | yes | yes | yes | yes | yes | yes | yes | yes | yes | yes | yes | yes | yes |
| TZ_A10 | yes | yes** | yes | yes | yes | yes | yes | yes | yes | yes | yes | yes | yes |
| TZ_A11 | yes | yes** | yes | yes | yes | yes | yes | yes | yes | yes | yes | yes | yes |

| # | Container and  Closure (yes / no) | | Trade / Brand name (* no ®, ** TM  instead of ®) | | Name of active  ingredient | | Manufacturer's name and logo (* no logo) | | Manufacturer's full address | | Medicine strength (mg/unit) | | Dosage form | | Number of units per container | | Dosage statement | | Batch/lot number | | Date of manufacture and expiry (* no date of manufacture) | | Storage information | | Leaflet or package insert | |
| --- | --- | --- | --- | --- | --- | --- | --- | --- | --- | --- | --- | --- | --- | --- | --- | --- | --- | --- | --- | --- | --- | --- | --- | --- | --- | --- |
| vetA1 | yes | | yes* | | yes | | yes | | yes | | yes | | yes | | yes | | yes | | yes | | yes | | yes | | yes | |
| vetA2 | yes | | yes* | | yes | | yes | | yes | | yes | | yes | | yes | | yes | | yes | | yes | | yes | | yes | |
| vetA3 | yes | | yes | | yes | | yes | | yes | | yes | | yes | | yes | | yes | | yes | | yes | | yes | | yes | |
| vetA4 | N/A | | N/A | | yes | | yes | | yes | | yes | | yes | | N/A | | yes | | yes | | yes | | yes | | N/A | |
| # | | Container and  Closure (yes / no) | | Trade / Brand name (* no ®, ** TM  instead of ®) | | Name of active  ingredient | | Manufacturer's name and logo (* no logo) | | Manufacturer's full address | | Medicine strength (mg/unit) | | Dosage form | | Number of units per container | | Dosage statement | | Batch/lot number | | Date of manufacture and expiry (* no date of manufacture) | | Storage information | | Leaflet or package insert |
| GH_M1 | | yes | | yes | | yes | | yes | | yes | | yes | | yes | | yes | | yes | | yes | | yes | | yes | | yes |
| GH_M2 | | yes | | yes | | yes | | yes | | yes | | yes | | yes | | yes | | yes | | yes | | yes | | yes | | yes |
| GH_M3 | | yes | | yes* | | yes | | yes | | yes | | yes | | yes | | yes | | yes | | yes | | yes | | yes | | yes |
| GH_M4 | | yes | | yes | | yes | | yes | | yes | | yes | | yes | | yes | | yes | | yes | | yes | | yes | | yes |
| BF/CI_M1 | | yes | | yes | | yes | | **no*** | | **no** | | yes | | yes | | yes | | yes | | yes | | yes | | yes | | yes |
| BF/CI_M2 | | yes | | **no** | | yes | | yes | | **no** | | yes | | yes | | N/A | | N/A | | yes | | **no*** | | N/A | | N/A |
| BF/CI_M3 | | N/A | | **no** | | yes | | yes | | yes | | yes | | yes | | N/A | | N/A | | yes | | yes | | yes | | N/A |
| BF/CI_M4 | | yes | | **no** | | yes | | **no** | | **no** | | yes | | yes | | yes | | yes | | yes | | yes | | yes | | yes |
| BF/CI_M5 | | yes | | yes* | | yes | | yes | | yes | | yes | | yes | | yes | | yes | | yes | | yes | | yes | | **no** |
| BF/CI_M6 | | yes | | yes | | yes | | yes | | **no** | | yes | | yes | | yes | | yes | | yes | | **no*** | | yes | | yes |
| BF/CI_M7 | | yes | | yes* | | yes | | **no*** | | yes | | yes | | yes | | yes | | yes | | yes | | yes | | yes | | yes |
| BF/CI_M8 | | yes | | yes* | | yes | | yes | | yes | | yes | | yes | | yes | | yes | | yes | | yes | | yes | | yes |
| TZ_M1 | | yes | | yes* | | yes | | yes | | yes | | yes | | yes | | yes | | yes | | yes | | yes | | yes | | N/A |
| TZ_M2 | | yes | | **no** | | yes | | yes | | N/A | | yes | | yes | | yes | | yes | | N/A | | N/A | | yes | | N/A |
| TZ_M3 | | yes | | yes* | | yes | | yes | | yes | | yes | | yes | | yes | | yes | | yes | | yes | | yes | | N/A |
| TZ_M4 | | yes | | yes | | yes | | yes | | yes | | yes | | yes | | yes | | yes | | yes | | yes | | yes | | yes |
| TZ_M5 | | yes | | yes | | yes | | yes | | yes | | yes | | yes | | yes | | yes | | yes | | yes | | yes | | yes |
| TZ_M6 | | yes | | yes | | yes | | **no*** | | yes | | yes | | yes | | yes | | yes | | yes | | **no*** | | yes | | yes |
| TZ_M7 | | yes | | yes * | | yes | | yes | | yes | | yes | | yes | | yes | | yes | | yes | | yes | | yes | | yes |
| TZ_M8 | | yes | | yes * | | yes | | yes | | yes | | yes | | yes | | yes | | yes | | yes | | yes | | yes | | yes |

| # | Container and  Closure (yes / no) | Trade / Brand name (* no ®, ** TM  instead of ®) | Name of active  ingredient | Manufacturer's name and logo (* no logo) | Manufacturer's full address | Medicine strength (mg/unit) | Dosage form | Number of units per container | Dosage statement | Batch/lot number | Date of manufacture and expiry (* no date of manufacture) | Storage information | Leaflet or package insert |
| --- | --- | --- | --- | --- | --- | --- | --- | --- | --- | --- | --- | --- | --- |
| GH_P1 | yes | **no** | yes | yes | N/A | yes | N/A | yes | yes | yes | **no*** | N/A | N/A |
| BF/CI_P1 | yes | yes | yes | yes | yes | yes | yes | yes | yes | yes | yes | yes | yes |
| TZ_P1 | yes | yes* | yes | yes | N/A | yes | yes | yes | yes | yes | yes | N/A | N/A |
| TZ_P2 | yes | yes** | yes | yes | yes | yes | yes | yes | yes | yes | **no*** | yes | N/A |
| TZ_P3 | yes | yes | yes | yes | yes | yes | yes | yes | yes | yes | yes | yes | N/A |
| TZ_P4 | yes | yes* | yes | yes | yes | yes | yes | yes | yes | yes | yes | yes | N/A |
| TZ_P5 | yes | **no** | yes | yes | yes | yes | yes | yes | yes | yes | yes | yes | yes |
