# Supplementary material for: Quality and composition of Albendazole, Mebendazole and Praziquantel available in Burkina Faso, Côte d’Ivoire, Ghana and Tanzania
Source: PLoS Negl Trop Dis. 2021 Jan 25;15(1):e0009038. doi: 10.1371/journal.pntd.0009038 (PMC7861518; doi:10.1371/journal.pntd.0009038)
Supplement: S3 Table — (DOCX) [file pntd.0009038.s003.docx]

**S3 Table:** **Visual examination of Albendazole, veterinarian Albendazole, Mebendazole and Praziquantel formulations** (**bold**: did not pass specifications)

| # | Uniformity of shape | Uniformity of size | Uniformity of colour | Uniformity of  texture | [Uniformity of]  Markings | Lack of breaks, cracks and splits | Lack of embedded surface spots or contamination | [Uniformity of] Smell |
| --- | --- | --- | --- | --- | --- | --- | --- | --- |
| GH_A1 | yes | yes | yes | **no** | yes | **no** | yes | yes |
| GH_A2 | yes | yes | yes | yes | yes | yes | yes | yes |
| GH_A3 | N/A | N/A | N/A | N/A | N/A | N/A | N/A | yes |
| GH_A4 | yes | yes | yes | yes | yes | yes | yes | yes |
| GH_A5 | yes | yes | yes | yes | yes | yes | yes | yes |
| GH_A6 | yes | yes | yes | yes | yes | yes | yes | yes |
| GH_A7 | yes | yes | yes | yes | yes | yes | **no** | yes |
| GH_A8 | yes | yes | yes | yes | yes | yes | yes | yes |
| GH_A9 | yes | yes | yes | yes | yes | yes | yes | yes |
| GH_A10 | yes | yes | yes | yes | yes | yes | yes | yes |
| GH_A11 | yes | yes | yes | yes | yes | yes | yes | yes |
| GH_A12 | yes | yes | yes | **no** | yes | yes | yes | yes |
| GH_A13 | yes | yes | yes | yes | yes | yes | yes | yes |
| BF/CI_A1 | yes | yes | yes | yes | yes | yes | yes | yes |
| BF/CI_A2 | yes | yes | yes | **no** | yes | yes | yes | yes |
| BF/CI_A3 | yes | yes | yes | yes | yes | yes | yes | yes |
| BF/CI_A4 | yes | yes | yes | yes | yes | **no** | yes | yes |
| BF/CI_A5 | yes | yes | yes | yes | yes | yes | yes | yes |
| BF/CI_A6 | yes | yes | yes | no | yes | **no** | **no** | yes |
| BF/CI_A7 | yes | yes | yes | yes | yes | yes | yes | yes |
| BF/CI_A8 | yes | yes | yes | yes | yes | yes | yes | yes |
| BF/CI_A9 | yes | yes | yes | yes | yes | yes | yes | yes |
| TZ_A1 | yes | yes | yes | yes | yes | yes | yes | yes |
| TZ_A2 | yes | yes | yes | yes | yes | yes | yes | yes |
| TZ_A3 | yes | yes | yes | yes | yes | yes | yes | yes |
| TZ_A4 | yes | yes | yes | yes | yes | yes | yes | yes |
| TZ_A5 | yes | yes | yes | yes | yes | yes | yes | yes |
| TZ_A6 | yes | yes | yes | yes | yes | yes | yes | yes |
| TZ_A7 | N/A | N/A | N/A | N/A | N/A | N/A | N/A | yes |
| TZ_A8 | yes | yes | yes | yes | N/A | yes | yes | yes |
| TZ_A9 | yes | yes | yes | yes | yes | yes | yes | yes |
| TZ_A10 | yes | yes | yes | yes | yes | yes | yes | yes |
| TZ_A11 | N/A | N/A | N/A | N/A | N/A | N/A | N/A | yes |

| # | Uniformity of shape | Uniformity of size | Uniformity of colour | Uniformity of  texture | [Uniformity of]  Markings | Lack of breaks, cracks and splits | Lack of embedded surface spots or contamination | [Uniformity of] Smell |
| --- | --- | --- | --- | --- | --- | --- | --- | --- |
| vetA1 | yes | yes | yes | yes | yes | yes | yes | yes |
| vetA2 | yes | yes | yes | yes | yes | yes | yes | yes |
| vetA3 | yes | yes | yes | yes | yes | **no** | yes | yes |
| vetA4 | yes | yes | yes | **no** | yes | yes | yes | yes |

| # | Uniformity of shape | Uniformity of size | Uniformity of colour | Uniformity of  texture | [Uniformity of]  Markings | Lack of breaks, cracks and splits | Lack of embedded surface spots or contamination | [Uniformity of] Smell |
| --- | --- | --- | --- | --- | --- | --- | --- | --- |
| GH_M1 | yes | yes | yes | yes | yes | yes | yes | yes |
| GH_M2 | yes | yes | yes | yes | yes | yes | yes | yes |
| GH_M3 | yes | yes | yes | **no** | yes | **no** | **no** | yes |
| GH_M4 | yes | yes | yes | yes | yes | yes | yes | yes |
| BF/CI_M1 | yes | yes | **no** | yes | yes | yes | yes | yes |
| BF/CI_M2 | yes | yes | yes | yes | yes | yes | yes | yes |
| BF/CI_M3 | yes | yes | yes | yes | yes | yes | yes | yes |
| BF/CI_M4 | yes | yes | yes | yes | yes | yes | yes | yes |
| BF/CI_M5 | yes | yes | yes | yes | yes | yes | yes | yes |
| BF/CI_M6 | yes | yes | yes | yes | yes | yes | yes | yes |
| BF/CI_M7 | yes | yes | yes | yes | yes | yes | yes | yes |
| BF/CI_M8 | yes | yes | yes | yes | yes | yes | yes | yes |
| TZ_M1 | yes | yes | yes | yes | yes | yes | yes | yes |
| TZ_M2 | yes | yes | yes | yes | yes | yes | yes | yes |
| TZ_M3 | yes | yes | yes | yes | N/A | yes | yes | yes |
| TZ_M4 | yes | yes | yes | yes | yes | yes | yes | yes |
| TZ_M5 | N/A | N/A | N/A | N/A | N/A | N/A | N/A | yes |
| TZ_M6 | yes | yes | yes | yes | yes | yes | yes | yes |
| TZ_M7 | yes | yes | yes | yes | yes | **no** | yes | yes |
| TZ_M8 | N/A | N/A | N/A | N/A | N/A | N/A | N/A | yes |

| # | Uniformity of shape | Uniformity of size | Uniformity of colour | Uniformity of  texture | [Uniformity of]  Markings | Lack of breaks, cracks and splits | Lack of embedded surface spots or contamination | [Uniformity of] Smell |
| --- | --- | --- | --- | --- | --- | --- | --- | --- |
| GH_P1 | yes | yes | yes | yes | yes | yes | yes | yes |
| BF/CI_P1 | yes | yes | yes | yes | yes | yes | yes | yes |
| TZ_P1 | yes | yes | yes | yes | yes | **no** | (yes) | yes |
| TZ_P2 | yes | yes | yes | yes | yes | yes | yes | yes |
| TZ_P3 | yes | yes | yes | yes | yes | yes | yes | yes |
| TZ_P4 | yes | yes | yes | yes | yes | **no** | (yes) | yes |
| TZ_P5 | yes | yes | yes | **no** | yes | yes | yes | yes |
