# Supplementary material for: Quality and composition of Albendazole, Mebendazole and Praziquantel available in Burkina Faso, Côte d’Ivoire, Ghana and Tanzania
Source: PLoS Negl Trop Dis. 2021 Jan 25;15(1):e0009038. doi: 10.1371/journal.pntd.0009038 (PMC7861518; doi:10.1371/journal.pntd.0009038)
Supplement: S4 Table — (DOCX) [file pntd.0009038.s004.docx]

**S4 Table: Pharmacopoeial analysis of Albendazole, veterinarian Albendazole, Mebendazole and Praziquantel** (**bold**: did not pass specifications)

| # | Brand name (batch number) | Labelled API content [mg] | Mass uniformity:  fraction of samples passed | Disintegration times  [min]  passed: ≤ 30 min | TLC results:  [yes / undercutting /  exceeding]  passed: between 80 % and 100 % l.c. | HPLC results:  API content [%] (RSD)  passed: between 90 % and 110 % l.c. | Dissolution results:  released API [%]  passed: Q ≥ 80 % |
| --- | --- | --- | --- | --- | --- | --- | --- |
| GH_A1 | Abee-400 | 400 | 100 % | 6.25 - 8.50 | yes | 92.1 (1.6) | **60.5** |
| GH_A2 | Albenaz | 400 | 100 % | **45+** | yes | **87.7 (1.4)** | **0.0** |
| GH_A3 | Albendazole oral suspension | 400/20 mL | N/A | N/A | (yes) | 95.7 | N/A |
| GH_A4 | Eskaben 400 | 400 | 100 % | **45+** | yes | 97.6 (1.1) | **0.0** |
| GH_A5 | Nesben | 200 | 100 % | 2.00 - 3.50 | yes | 95.4 (3.6) | 93.0 |
| GH_A6 | Sequizol (AT97) | 400 | 100 % | 24.25 - 29.25 | yes | 92.9 | **53.9** |
|  | Sequizol (AT01) |  |  |  | yes | 94.6 | **48.7** |
| GH_A7 | Tacizol | 400 | 100 % | **45+** | yes | **87.2 (2.3)** | **1.1** |
| GH_A8 | Tanzol | 400 | 100 % | 10.50 - 12.00 | yes | **87.3 (1.4)** | 85.0 |
| GH_A9 | Wormbat-400 | 400 | 100 % | 3.75 - 6.25 | **undercutting** | **74.1 (3.5)** | **41.6** |
| GH_A10 | Wormplex 400 (WPTP0038) | 400 | 100 % | 11.00 - 25.50 | yes | 100.7 (0.9) | 89.3 |
|  | Wormplex 400 (WPTP0039) |  |  |  | yes | 98.0 (3.8) | 92.9 |
| GH_A11 | Wormron 400 (WA1806) | 400 | 90 % | **38.25 - 46.75** | yes | 93.1 (1.6) | **12.8** |
|  | Wormron 400 (WA1801) |  |  | **23.25 - 32.25** | yes | 98.3 (0.1) | **22.0** |
| GH_A12 | Wormzap | 400 | 100 % | 3.50 - 4.50 | yes | **84.6 (13.5)** | 84.5 |
| GH_A13 | Zentel | 200 | 100 % | 8.75 - 9.75 | yes | 99.3 (1.6) | 98.9 |
| BF/CI_A1 | ABZ | 400 | 100 % | 20.75 - 23.25 | yes | **84.1 (1.4)** | **77.5** |
| BF/CI_A2 | Albendazole TM | 400 | 100 % | 2.00 - 2.75 | yes | 91.8 (2.2) | 92.0 |
| BF/CI_A3 | Bendex-400 | 400 | 100 % | **45+** | yes | 97.2 (0.3) | **0.0** |
| BF/CI_A4 | Elband 400 | 400 | 100 % | 11.25 - 13.25 | yes | 94.3 | **56.2** |
| BF/CI_A5 | Lyben | 400 | N/A | 15.25 - 16.50 | yes | 91.5 | **14.3** |
| BF/CI_A6 | Sanozol | 400 | 100 % | **45+** | yes | 92.0 (1.8) | **3.4** |
| BF/CI_A7 | Tanizol | 400 | 100 % | 10.50 - 24.25 | yes | 95.2 (3.6) | **64.7** |
| BF/CI_A8 | Verex (186) | 400 | **86.7 %** | **45+** | **exceeding** | 100.7 (2.7) | **0.4** |
|  | Verex (175) |  |  |  | yes | 95.4 | **0.2** |
| BF/CI_A9 | Verzol (TE-6717) | 400 | 100 % | 4.00 - 4.25 | yes | **86.4 (2.0)** | 97.0 |
|  | Verzol (TE-6677) |  |  |  | yes | 96.7 | 99.1 |
|  | Verzol (TE-6716) |  |  |  | yes | 95.4 (0.5) | 97.8 |
| TZ_A1 | Alben (180018) | 200 | 95.8 % | **35.75 - 49.75** | yes | 99.8 | **68.4** |
|  | Alben (170023) |  |  |  | yes | 98.3 (2.7) | 81.0 |
|  | Alben (180012) |  |  |  | yes | 95.1 | **70.3** |
| TZ_A2 | Albendazole 400mg | 400 | 100 % | 7.25 - 7.75 | yes | 92.4 (5.0) | 96.0 |
| TZ_A3 | Albi | 400 | 100 % | 1.00 - 1.25 | yes | 94.5 | 97.7 |
| TZ_A4 | Alzental (ALZET S002) | 400 | 100 % | **37.50 - 45+** | yes | **89.9 (1.4)** | 97.8 |
|  | Alzental (ALZET S001) |  |  | 12.50 - 14.25 | yes | **86.3** | N/A |
| TZ_A5 | Anthel (BV6006) | 400 | 100 % | 19.50 - 23.75 | yes | 100.1 | **70.9** |
|  | Anthel (BV7004) |  |  |  | yes | 98.6 (1.8) | 83.4 |
| TZ_A6 | Azentel (360766) | 400 | 100 % | 6.25 - 12.25 | yes | 98.0 | 100.5 |
|  | Azentel (350579) |  |  |  | yes | 92.4 | N/A |
|  | Azentel (370425) |  |  |  | yes | 100.4 (0.8) | 95.5 |
| TZ_A7 | Benpham suspension | 400/10 mL | N/A | N/A | (yes) | 93.5 (0.8) | N/A |
| TZ_A8 | Elyzole (5I07) | 200 | 100 % | **45+** | yes | 99.8 (1.4) | **24.1** |
|  | Elyzole (7G131) |  |  |  | yes | 104.0 (0.4) | **10.0** |
|  | Elyzole (6K01) |  |  |  | yes | 101.4 (1.2) | **26.1** |
| TZ_A9 | Womiban (FWX1706) | 400 | 100 % | 1.00 - 2.00 | yes | 94.9 (0.9) | **53.9** |
|  | Womiban (FWX1701) |  |  |  | yes | 92.1 (3.6) | **40.4** |
| TZ_A10 | Zentel | 400 | 100 % | N/A | yes | 97.2 | 97.1 |
| TZ_A11 | Zentel suspension | 400/20 mL | N/A | N/A | (yes) | 104.3 (0.6) | N/A |

| # | Brand name (batch numbers) | Labelled API content [mg] | Mass uniformity:  fraction of samples passed | Disintegration times  [min]  passed: ≤ 30 min | TLC results:  [yes / undercutting /  exceeding]  passed: between 80 % and 100 l.c. | HPLC results:  API content [%] (RSD)  passed: between 90 % and 110 % l.c. | Dissolution results:  released API [%]  passed: Q ≥ 80 % |
| --- | --- | --- | --- | --- | --- | --- | --- |
| vetA1 | Albendafarm 1500 | 1500 | 100 % | 2.75 - 3.75 | yes | N/A | N/A |
| vetA2 | Albendafarm 2500 | 2500 | 100 % | 2.00 - 3.25 | yes | N/A | N/A |
| vetA3 | Ashialben 300 | 300 | 100 % | 10.25 - 15.75 | **undercutting** | N/A | N/A |
| vetA4 | Ashialben 600 | 600 | 100 % | N/A | **undercutting** | N/A | N/A |

| # | Brand name (batch number) | Labelled API content [mg] | Mass uniformity:  fraction of samples passed | Disintegration times  [min]  passed: ≤ 30 min | TLC results:  [yes / undercutting /  exceeding]  passed: between 80 % and 100 % l.c. | HPLC results:  API content [%] (RSD)  passed: between 90 % and 110 % l.c. | Dissolution results  released API [%]  Int. Ph. passed: Q ≥ 60 % in100 mg gives; Q ≥ 70 % in 500 mg gives  (USP passed: Q ≥ 75 %) |
| --- | --- | --- | --- | --- | --- | --- | --- |
| GH_M1 | De Wome 500 | 500 | 90 % | 6.75 - 8.25 | **undercutting** | 101.2 (4.3) | **35.0** |
| GH_M2 | Mentel | 500 | 90 % | **45+** | **exceeding** | 97.8 (5.6) | **19.4** |
| GH_M3 | Trazole-500 | 500 | **33.3 %** | 8.75 - 17.75 | **exceeding** | 101.6 (7.6) | 87.0 |
| GH_M4 | Vermox | 500 | 100 % | 6.25 - 7.50 | **exceeding** | 102.8 (0.5) | 108.5 |
| BF/CI_M1 | Carben | 100 | 94.4 % | **45+** | **exceeding** | 106.4 (0.6) | **19.5** (N/A) |
| BF/CI_M2 | Mébendazole (G07002) | 100 | 100 % | 2.00 - 5.25 | yes | 104.5 (1.1) | **27.2 (73.1)** |
| BF/CI_M3 | Mebendazole (F0164) | 100 | 100 % | 1.00 - 1.50 | **exceeding** | 101.3 (1.3) | **27.4** (103.0) |
| BF/CI_M4 | Mebendazole (MZ-1811) | 100 | 95 % | 15.75 - 19.25 | yes | 90.0 (2.3) | **29.0** (92.3) |
| BF/CI_M5 | Nebenda | 100 | 90 % | **45+** | **exceeding** | 98.2 (3.2) | **1.0 (14.9)** |
| BF/CI_M6 | Oziben (DOR 1702) | 100 | 100 % | 3.75 - 9.00 | **exceeding** | 101.5 (1.7) | **27.0** (90.5) |
|  | Oziben (DOR 1701) | 100 |  |  | **exceeding** | 98.5 | **34.5** (N/A) |
| BF/CI_M7 | T-Medazol | 500 | 100 % | 0.25 - 0.75 | yes | 100.2 (2.8) | **69.1** |
| BF/CI_M8 | Wormin 500 (R70E8001) | 500 | 100 % | 1.75 - 2.00 | **exceeding** | 100.6 (0.5) | 88.9 |
|  | Wormin 500 (R70E8003) | 500 |  |  | yes | 100.1 | N/A |
| TZ_M1 | Astazole (254) | 100 | **83.3 %** | 0.75 - 2.50 | **exceeding** | 96.2 (1.2) | **25.4** (N/A) |
|  | Astazole (257) | 100 |  |  | **exceeding** | 98.8 (3.0) | **34.4** (N/A) |
|  | Astazole (256) | 100 |  |  | **exceeding** | 99.3 (3.1) | **24.2** (97.4) |
| TZ_M2 | Mebendazole BP 500mg | 500 | 100 % | 1.25 - 1.75 | **exceeding** | **78.7 (3.5)** | N/A |
| TZ_M3 | Mebrone-100 | 100 | **70 %** | 0.75 - 5.75 | **exceeding** | 97.4 (1.7) | **29.1** (N/A) |
| TZ_M4 | Natoa (71356) | 100 | 100 % | **45+** | **exceeding** | 101.5 (2.8) | **1.4** (N/A) |
|  | Natoa (69900) | 100 |  |  | **exceeding** | 100.8 (0.7) | **0.0 (3.42)** |
| TZ_M5 | Natoa suspension | 600/30 mL | N/A | N/A | N/A | 92.0 (1.4) | N/A |
| TZ_M6 | Vermox | 500 | 100 % | N/A | **exceeding** | 94.0 | N/A |
| TZ_M7 | Wormnil | 100 | 100 % | 1.00 - 1.50 | yes | 100.4 (0.6) | **32.1** (96.5) |
| TZ_M8 | Wormol suspension | 600/30 mL | N/A | N/A | N/A | 94.8 (0.3) | N/A |

| # | Brand name (batch number) | Labelled API content [mg] | Mass uniformity:  fraction of samples passed | Disintegration times [min]  passed: ≤ 30 min | TLC results:  [yes / undercutting /  exceeding]  passed: between 80 % and 100 % l.c. | HPLC results:  API content [%] (RSD)  passed: between 90 % and 110 % l.c. | Dissolution results:  released API [%]  passed: Q ≥ 75 % |
| --- | --- | --- | --- | --- | --- | --- | --- |
| GH_P1 | Praziquantel 600 | 600 | 100 % | 2.50 - 4.75 | yes | 101.5 (0.9) | **70.0** |
| BF/CI_P1 | Biltricide | 600 | 100 % | N/A | yes | 106.5 | 90.2 |
| TZ_P1 | Bermoxel (A8AO3) | 600 | 100 % | 1.00 - 2.25 | yes | 96.7 (1.0) | **59.2** |
|  | Bermoxel (N/A) |  |  |  | yes | 101.2 (0.6) | **69.7** |
| TZ_P2 | Cesol | 600 | 100 % | 8.75 - 15.25 | yes | 98.9 (3.5) | 81.2 |
| TZ_P3 | Distocide | 600 | 100 % | 8.00 - 10.25 | yes | 96.2 (2.5) | 90.1 |
| TZ_P4 | Prazikant (BZ7034) | 600 | **75 %** | 2.00 - 5.75 | yes | 91.8 (5.5) | 80.3 |
|  | Prazikant (N/A) |  |  |  | yes | 97.6 (5.3) | 75.6 |
| TZ_P5 | Praziquantel-600 (140007) | 600 | 100 % | 5.50 - 16.00 | yes | 99.6 | **73.7** |
|  | Praziquantel-600 (160004) |  |  |  | yes | N/A | N/A |
|  | Praziquantel-600 (170003) |  |  | **24.75 - 35.50** | yes | 93.4 (1.0) | **47.3** |
